# Highly efficient ammonia synthesis at low temperature over a Ru–Co catalyst with dual atomically dispersed active centers†

**DOI:** 10.1039/d1sc00304f

**Published:** 2021-04-07

**Authors:** Xuanbei Peng, Han-Xuan Liu, Yangyu Zhang, Zheng-Qing Huang, Linlin Yang, Yafei Jiang, Xiuyun Wang, Lirong Zheng, Chunran Chang, Chak-tong Au, Lilong Jiang, Jun Li

**Affiliations:** National Engineering Research Center of Chemical Fertilizer Catalyst, Fuzhou University Fuzhou Fujian 350002 China xywang2017@fzu.edu.cn jllfzu@sina.cn jll@fzu.edu.cn; Shaanxi Key Laboratory of Energy Chemical Process Intensification, School of Chemical Engineering and Technology, Xi'an Jiaotong University Xi'an 710049 China; Department of Chemistry, Southern University of Science and Technology Shenzhen China; Institute of High Energy Physics, Chinese Academy of Sciences Beijing China; Department of Chemistry, Tsinghua University Beijing China

## Abstract

The desire for a carbon-free society and the continuously increasing demand for clean energy make it valuable to exploit green ammonia (NH_3_) synthesis that proceeds *via* the electrolysis driven Haber–Bosch (eHB) process. The key for successful operation is to develop advanced catalysts that can operate under mild conditions with efficacy. The main bottleneck of NH_3_ synthesis under mild conditions is the known scaling relation in which the feasibility of N_2_ dissociative adsorption of a catalyst is inversely related to that of the desorption of surface N-containing intermediate species, which leads to the dilemma that NH_3_ synthesis could not be catalyzed effectively under mild conditions. The present work offers a new strategy *via* introducing atomically dispersed Ru onto a single Co atom coordinated with pyrrolic N, which forms RuCo dual single-atom active sites. In this system the d-band centers of Ru and Co were both regulated to decouple the scaling relation. Detailed experimental and theoretical investigations demonstrate that the d-bands of Ru and Co both become narrow, and there is a significant overlapping of t_2g_ and e_g_ orbitals as well as the formation of a nearly uniform Co 3d ligand field, making the electronic structure of the Co atom resemble that of a “free-atom”. The “free-Co-atom” acts as a bridge to facilitate electron transfer from pyrrolic N to surface Ru single atoms, which enables the Ru atom to donate electrons to the antibonding π* orbitals of N_2_, thus resulting in promoted N_2_ adsorption and activation. Meanwhile, H_2_ adsorbs dissociatively on the Co center to form a hydride, which can transfer to the Ru site to cause the hydrogenation of the activated N_2_ to generate N_2_H_*x*_ (*x* = 1–4) intermediates. The narrow d-band centers of this RuCo catalyst facilitate desorption of surface *NH_3_ intermediates even at 50 °C. The cooperativity of the RuCo system decouples the sites for the activation of N_2_ from those for the desorption of *NH_3_ and *N_2_H_*x*_ intermediates, giving rise to a favorable pathway for efficient NH_3_ synthesis under mild conditions.

## Abstract 2

The desire for a carbon-free society and the continuously increasing demand for clean energy make it valuable to exploit green ammonia (NH_3_) synthesis that proceeds *via* the electrolysis driven Haber–Bosch (eHB) process.

## Graphical abstract

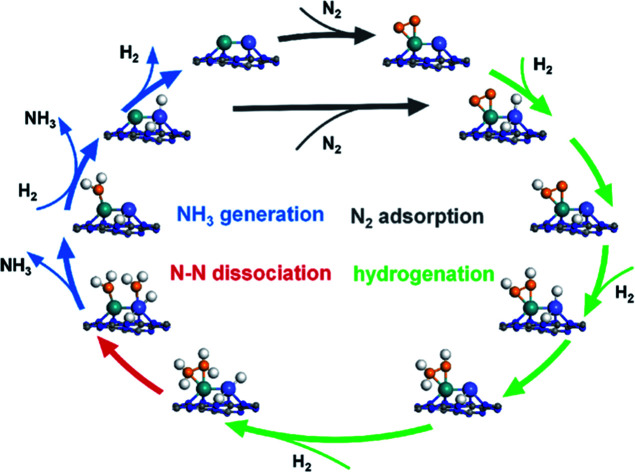

## Introduction

The N_2_-to-NH_3_ conversion is one of the most important reactions for human society. NH_3_ is not only used for the production of fertilizers and important chemicals, but also considered as a future fuel alternative and hydrogen storage vector.^1–7^ The potential utilization of NH_3_ in the coming chemical industry revolution is highly promising.^8^ Unfortunately, the industrial synthesis of NH_3_*via* the Haber–Bosch (HB) process using iron-based catalysts requires stringent reaction conditions (400–600 °C, 20–40 MPa),^9–12^ and consumes 1–2% of the global energy and produces ∼1.5 tons of carbon dioxide per ton of NH_3_.^10–12^ Traditionally, the H_2_ supply for the HB process is mainly from coal or natural gas through the water-gas shift (WGS) or methane reforming reaction,^13^ and these processes account for the energy requirement and carbon dioxide production. Therefore, it is imperative to develop new HB technology that is both environmentally friendly and energy saving. Recently, with the green generation of electricity using renewable sources (such as hydro, wind, solar and tidal), it becomes economically acceptable to use the H_2_ produced by water electrolysis, and NH_3_ can be synthesized from renewable H_2_ and N_2_*via* the electrolysis driven Haber–Bosch (eHB) process.

Nowadays, the subsequent NH_3_ re-conversion to H_2_ and the handling as well as shipping infrastructure including regulations for transportation are already in place.^6^ The above process follows a protocol of “renewable power supply → electrolytic H_2_ production → NH_3_ synthesis → NH_3_ storage → H_2_ energy”, in which efficient utilization of renewable energy for green production of NH_3_ could be realized to achieve a “zero-loss” seasonal energy storage cycle that is carbon-free.^3,13–15^ However, the present bottleneck is that NH_3_ cannot be synthesized under mild conditions. Currently, huge plants are required, and for a plant with an annual capacity of 200 000 tons, the investment is at the scale of US$ 1000–2000 per ton of ammonia.^7^ The aim is to match the pressure of the NH_3_ synthetic system with that of the electrolysis system employed for H_2_ production (<5 MPa, mostly lying in the range of 1.0–3.2 MPa) so as to avoid expensive pressure ramping.^15^ Therefore, advanced catalysts that are adoptable to an efficient eHB process are urgently needed, which can activate N_2_ for NH_3_ synthesis at lower pressure.

The major obstacle of NH_3_ synthesis under mild conditions is the scaling relation.^15–18^ In order to achieve NH_3_ synthesis under mild conditions, great efforts have been devoted to seek catalytic materials that can either lower N_2_ activation barriers^19–21^ or circumvent the scaling relationship.^5,6,22–27^ Previous advanced surface-science and theoretical investigations disclose that d-block transition metals (TMs), such as Re, Mo, Ru, Co and Rh, obey the scaling relationship because their surface d-bands are broad in bulk metals.^18,28–31^ The d-band center of such metals results in strong binding of N-containing intermediates, which require high temperature to desorb. Upon regulating the surface d-band centers of TMs, it is possible to simultaneously tune the binding energy of N_2_ adsorption and that of surface N-containing intermediates. By so doing, the scaling relation for TMs can be decoupled. Indeed, the surface d-band of a metal-based catalyst can be effectively tuned through the strategy of single-atom catalysts (SACs)^32–34^ or single-cluster catalysts (SCCs),^23,35^ leading to d-band adsorption modes different from those of pure metals and conventional alloy compositions.^36,37^ The unusual electronic behavior of SACs and SCCs could also change the adsorption properties and bonding abilities of each metal atom,^30,38^ resulting in significant deviations from the scaling relationship.^36,39,40^ Among the SCCs, dual single-atom catalysts (DSACs) offer the simplest active sites for catalytic reactions.

For the well-known active Ru and Co metals in NH_3_ synthesis, the Co d-band is narrower than that of Ru because of the quantum primogenic effect,^41^ resulting in the N_2_ dissociative adsorption energy of Co being much lower than that of Ru. Despite the fact that the state-of-the-art Ru metal catalysts are efficient for NH_3_ synthesis, the loading and high cost limit the large-scale use of the noble metal. It is hence advantageous to achieve efficient utilization of Ru by having a small amount of Ru atomically dispersed on atomistic Co to generate a RuCo DSAC. It is expected that the surface d-bands of the two metal atoms could be regulated through the formation of a DSAC, resulting in unique electronic and/or geometric features different from those of monometallic Ru and Co metals.^42–44^ As there is so far no experimental demonstration on the use of DSACs for NH_3_ synthesis, we are interested in exploring whether the RuCo DSAC is capable of decoupling the scaling relationship and which mechanism this DSAC system might adopt. Especially, the following issues deserve investigation: (1) the existing form of dual active centers, (2) the state of the surface d-band, (3) the pathway of N_2_ activation and dissociation, and (4) how to decouple the scaling relation.

Herein, we report the preparation of a RuCo DSAC by introducing a Ru atom on a surface layer of Co–N–C material with Co atoms coordinated by a pyrrolic N of g-C_3_N_4_. It is found that with Ru atomically anchored on the surface of single-site Co, there is significant overlapping of Co t_2g_ and e_g_ orbitals, resulting in an electronic structure resembling that of a “free” Co atom. Our experimental and theoretical studies demonstrate that the cooperativity of Ru and Co dual single-atom active centers could decouple the scaling relationship by separating N_2_ adsorption and activation (on the Ru sites) from H_2_ dissociative adsorption as well as N-containing intermediate species (mainly *N_2_H_4_ and *NH_3_) desorption (on the Co sites). By so doing, the scaling relation over RuCo DSAC in NH_3_ synthesis can be decoupled, and the developed RuCo DSAC could efficiently promote NH_3_ synthesis at 200 °C, giving an NH_3_ synthesis rate of up to 1.24 mmol_NH_3__ g_cat_^−1^ h^−1^. We believe the acquired understanding can help to accomplish rational design of transition-metal-based DSACs for the decoupling of the scaling relation to achieve high catalytic efficiency under mild conditions as well as to develop advanced catalysts to achieve pressure matching between the electrolysis and catalytic systems in eHB technology.

## Results

### Confirmation of Ru and Co atomic dispersion

**Fig. 1:**
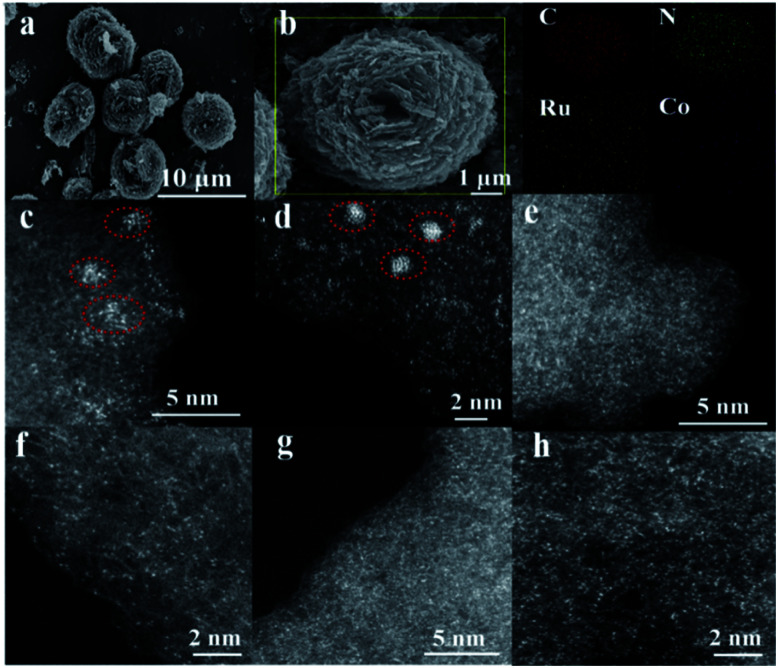
The morphology of the synthesized catalysts. (a) SEM image of RuCo DSAC, (b) SEM-mapping of N–K, C–K, Co–K and Ru–L over RuCo DSAC, (c–h) AC-STEM images: (c and d) Ru/N–C, (e and f) Co SAC and (g and h) RuCo DSAC samples.

To highlight the effect of the DSAC active centers, monometallic Ru/N–C and Co/N–C (denoted hereinafter as Co SAC) were also prepared for comparison purposes. Representative scanning electron microscopy (SEM) images demonstrate that the morphology of RuCo DSAC (Fig. 1a) is similar to that of Ru/N–C (Fig. S1a†) and Co SAC (Fig. S1b†), showing spherical structures of different sizes. The low magnification SEM-mapping of RuCo DSAC (Fig. 1b) reveals a homogeneous distribution of Ru and Co throughout the support material. And the amount of Ru and Co was 0.92 wt% and 2.24 wt%, respectively, according to the inductively coupled plasma atomic emission spectroscopy (ICP-AES) results (Table S1†). Structural information on RuCo DSAC was obtained based on X-ray diffraction (XRD) analysis. The characteristic peak at 26.4° (Fig. S2†) can be ascribed to the g-C_3_N_4_ phase. With the insertion of Ru and/or Co atoms into g-C_3_N_4_, this peak decreases in intensity, revealing a distorted g-C_3_N_4_ crystal structure. No obvious diffraction peaks ascribable to Ru and/or Co species can be discerned. Also, the transmission electron microscopy (TEM, Fig. S3a–d†) images of RuCo DSAC show no obvious Ru or Co nanoparticles (NPs). These reveal that the Ru and/or Co species of Co SAC and RuCo DSAC are highly dispersed either as tiny clusters or as single atoms. This conclusion is further determined from spherical aberration-corrected high-angle annular dark-field scanning transmission electron microscopy (AC-STEM) characterization.

According to the AC-STEM results, there is distribution of Ru single atoms as well as small clusters on Ru/N–C (Fig. 1c and d, and S4a–d†); the latter is a result of Ru aggregation as indicated by the individual bright dots of different intensity levels. Moreover, the AC-STEM images (Fig. 1e and f, and S5a–d†) taken at different regions of Co SAC show the predominant presence of Co single atoms (Fig. 1e and f, and S5a–d, related descriptions are provided in the ESI†). Interestingly, RuCo DSAC exhibits many individual bright dots (Fig. 1g and h). The dots over RuCo DSAC are repeatedly found at different magnifications (Fig. S6a–c†). Most of these dots show no obvious difference (Fig. 1g and h), and the results show that Ru and Co are uniformly dispersed. The result agrees with the fact that there is formation of dual single-atom Ru and Co as confirmed by extended X-ray absorption fine structure (EXAFS) measurements over RuCo DSAC. The conclusion is in accordance with the fact that there is a Ru–Ru bond attributable to Ru clusters as revealed by EXAFS measurements over Ru/N–C (Fig. S7†).

**Fig. 2:**
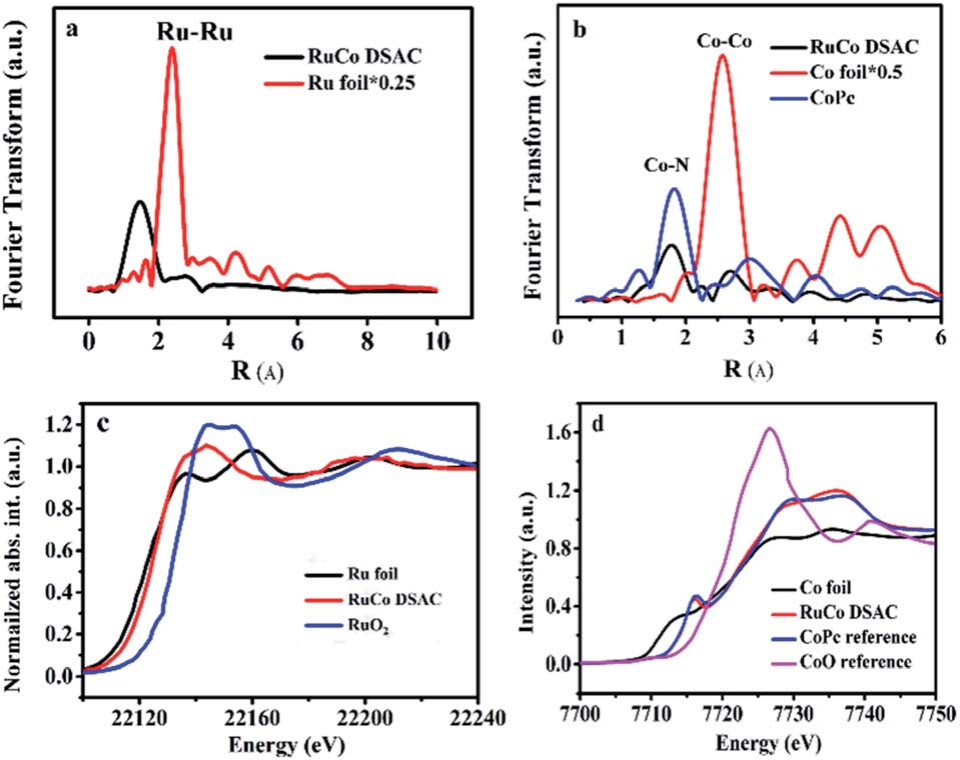
Physical characterization. (a) Ru K-edge EXAFS spectra of RuCo DSAC and reference samples, (b) Co K-edge EXAFS spectra over RuCo DSAC, (c) normalized Ru K-edge XANES spectra and (d) normalized Co K-edge XANES spectra over RuCo DSAC and reference samples.

The EXAFS Ru K-edge (Fig. 2a) and Co K-edge (Fig. 2b and S8†) spectra show no obvious Ru–Ru or Co–Co interaction over RuCo DSAC and Co SAC (Fig. S9†), providing direct evidence for the presence of individually isolated Ru and/or Co atoms in the samples. Assignment of signals from 1 to 4 Å in RoCo DSAC is further confirmed by detailed WT-EXAFS wavelet transform plots (Fig. S10†). A WT intensity maximum near 4.5 Å^−1^ can be assigned to the Co–N contribution. Moreover, the EXAFS fitting curves (Fig. S11a–d†) show that Ru (Fig. S11b†) and Co (Fig. S11d†) atoms over RuCo DSAC are coordinated with nitrogen atoms, and the coordination numbers (CN) of Ru–N and Co–N over RuCo DSAC are 2.9 ± 0.5 and 2.9 ± 0.9 (Table S2†), respectively, while the CN for Co–Ru is around 2.1.

We then performed XPS and XANES measurements to investigate the chemical state of Ru and Co species. XPS study results show that the surface Ru content in the case of RuCo DSAC is close to the total Ru content (total Ru content is determined by ICP-AES measurements). Notably, there is no detection of the Ru 3p signal after deep etching by Ar^+^ (Table S3†). These results indicate that Ru mainly disperses on the surface of DSAC. Moreover, the energy absorption edge of Ru K-edge XANES over RuCo DSAC (Fig. 2c) is higher than that of Ru foil but lower than that of RuO_2_, suggesting an oxidation state of Ru^*n*+^ (0 < *n* < 4) rather than Ru^4+^ or Ru^0^.^45^ Similarly, the Co K-edge XANES spectra (Fig. 2d) show that the absorption edge position of RuCo DSAC locates between that of Co foil and CoO bulk, suggesting that the single Co atom carries positive charge, which is in accord with the XPS Co 2p study (Fig. S12a†). The XPS binding energy (BE) of the Co 2p_3/2_ peak (Fig. S12a†) over RuCo DSAC is higher than that of Co^2+^ (779.2 eV), and lower than that of Co^3+^ (781.0 eV),^46^ suggesting that the surface Co atom has a hetero-valence state of Co^2+^ and Co^3+^. Additionally, the absorption edge of Ru K-edge over RuCo DSAC (Fig. S13†) is lower than that of Ru/N–C, while the BE of the XPS Co 2p_3/2_ peak for RuCo DSAC is higher than that for Co SAC (Fig. S12a†), indicating that there is electron transfer from Co to Ru *via* the Ru–Co bond,^47^ plausibly due to the higher electronegativity of the Ru atom.^48^ Meanwhile, the XPS Co 2p peaks of the RuCo DSAC sample significantly shift to lower BE values (−1.58 ± 0.7 eV, Fig. S12b†) when it is subject to Ar^+^ etching at different depths. These observations further suggest electron redistribution *via* electron transfer from Co to the surface Ru atom.

### “Free-Co-atom” and electron transfer

**Fig. 3:**
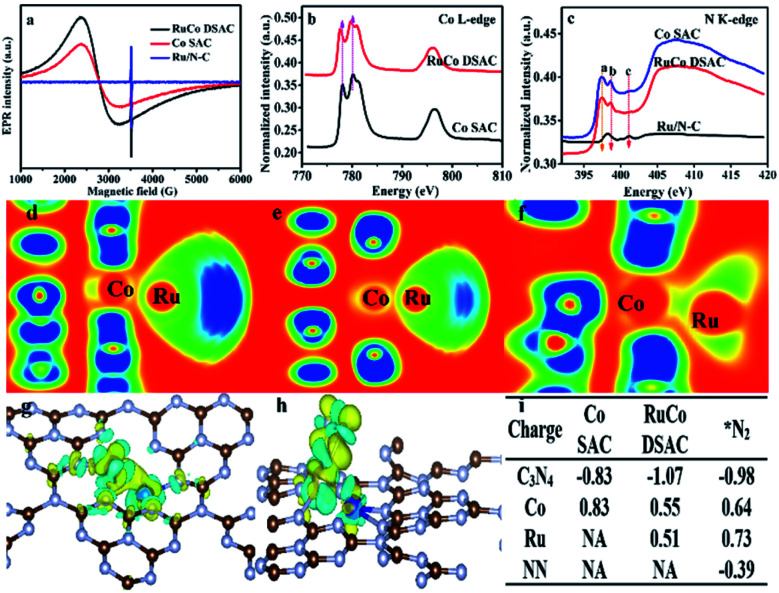
Evidence of charge transfer. (a) EPR spectra, (b and c) NEXAFS results: (b) Co L-edge and (c) N K-edge. (d–f) Electronic function location (EFL) for Ru and Co atoms on (d) graphitic N, (e) pyridinic N and (f) pyrrolic N species. (g and h) Charge density differences over (g) RuCo DSAC and (h) RuCo DSAC@N_2_. (i) Charge variation during N_2_ adsorption on RuCo DSAC and Co SAC samples.

Additional evidence of electron transfer between Co and Ru can be obtained by electron paramagnetic resonance (EPR) and NEXAFS analyses. According to the EPR spectra of Fig. 3a, the *g* value (3.0) of RuCo DSAC can be ascribed to an unpaired electron in the 3d_*x*^2^−*y*^2^_ orbital of Co^II^ and Ru^III^.^49^ In comparison with Co SAC, the value shift in *g* value and the shape broaden asymmetrically for RuCo DSAC, indicative of a dipolar broadening due to electron–electron interaction between atomically dispersed Ru and Co, and the phenomenon supports the idea of charge transfer,^50^ in agreement with the observation of Co K-edge NEXAFS results (Fig. 3b). It is to be noted that there is no EPR signal of Ru/N–C because of no obvious electron transfers between the Ru and N atoms. The Co L-edge NEXAFS over RuCo DSAC and Co SAC can be fitted into L2 and L3 sub-bands (Fig. 3b), matching well with the transition from 2p_1/2_ and 2p_3/2_ levels to the vacant d band,^51,52^ respectively. Compared with Co SAC, the absorption Co L-edge of RuCo DSAC shifts towards lower excitation energy by 0.6 eV. Meanwhile, the Co L-edge intensity of RuCo DSAC is lower than that of Co SAC (Fig. 3b), implying that the former has higher occupancy of Co 3d electrons. It is to be noted that the extent of electron transfer is dependent on the nature of surface unoccupied Co 3d charge, which could be semi-quantitatively calculated on the basis of the Co L-edge NEXAFS spectra (Fig. S14†), using the calculation method of Mattheiss and Dietz (see the ESI†).^53^ The relative number of unoccupied Co 3d charge (Kh_T_) in RuCo DSAC (14.7) is higher than that of Co SAC (11.2). The much higher unpaired Co 3d charge of RuCo DSAC indicates the higher feasibility of d electron donation from Co to the unoccupied Ru 3d t_2g_ (feature L2) and e_g_ (feature L3) states in the RuCo DSAC case.^36^

Owing to the fact that the total electron yield (TEY) is highly surface sensitive, we collected the N K-edge NEXAFS spectra of RuCo DSAC, Co SAC and Ru/N–C to gain insight into the direction of electron transfer (Fig. 3c). Over RuCo DSAC in the range of 397–402 eV, the characteristic π* peaks at 397.5 (peak *a*), 398.6 (peak *b*) and 401.2 (peak *c*) eV can be assigned to pyridinic, pyrrolic and graphitic nitrogen species, respectively.^54,55^ The presence of the π* peak is attributed to N coordination that involves electron transfer from N to the coordinated metal atom. Based on DFT calculation results including the electron localization function (ELF) (Fig. 3d–f), charge density differences (Fig. 3g and h), and Bader charges (Fig. 3i), it can be confirmed that there is charge transfer from the N atom of g-C_3_N_4_ to the Co atom, with loss of negative charge in the former while gain of negative charge in the latter. Then, the Co atom enriched with an electron acts as a bridge to facilitate the transfer of electrons from Co 3d orbitals to the Ru atom.

The extent of electron transfer from Co to Ru is dependent on the nature of the N species involved in the interaction. The chunk in ELF mapping (Fig. 3d–f) demonstrates the electron interaction between Co and Ru, and that the involvement of pyrrolic N species is the strongest (a greener chunk represents weaker electron interaction). The transferred electron density from the nitrogen species to Co and then to Ru follows the order pyrrolic N ≫ graphitic N > pyridinic N species (Table S4†). Taking together the N K-edge NEXAFS results (Fig. 3c), because of the absence of the *a* peak over Ru/N–C, it is deduced that Ru does not coordinate with pyrrolic N. In a previous study of ours, it was confirmed that single cobalt atoms can anchor and get stabilized on pyrrolic N species.^56^ Then, Co acts as a bridge to facilitate electron transfer from g-C_3_N_4_ through pyrrolic N and Co to the surface Ru atom. Consequently, there is a decrease of N_2_ adsorption energy, and the N_2_ molecule adsorbed on Ru shows a negative charge of −0.39 e^−^ (Fig. 3i). As a site for N_2_ adsorption, the electron-enriched Ru atom readily donates electrons to the antibonding π* orbitals of adsorbed N_2_, promoting N_2_ activation *via* the weakening of the N≡N triple bond.

**Fig. 4:**
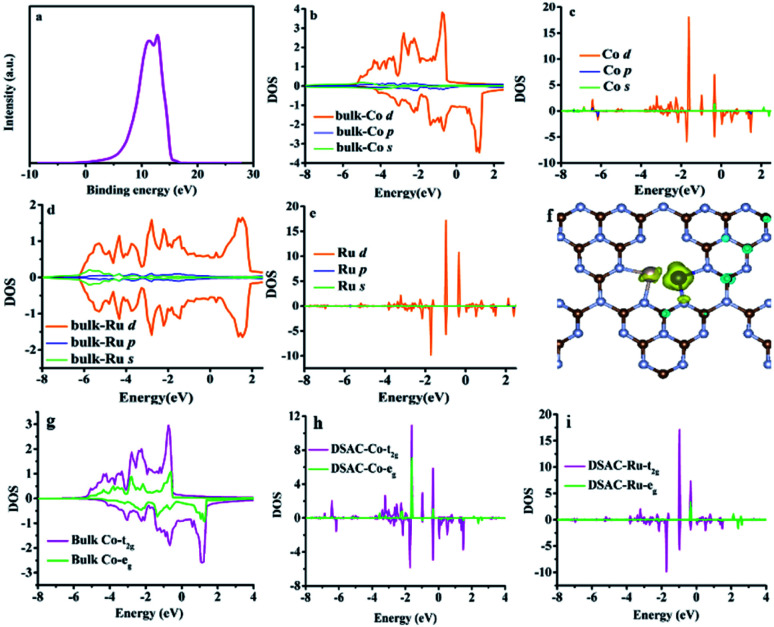
UPS and PDOS results. (a) UPS spectrum. Calculated s, p, and d-bands over (b) Co bulk, (c) Co in RuCo DSAC, (d) Ru bulk and (e) Ru in RuCo DSAC. (f) Spin density of RuCo DSAC. (g and h) t_2g_ and e_g_ for (g) Co bulk, (h) Co over RuCo DSAC and (i) Ru over RuCo DSAC.

The electron release and/or donation behaviors of RuCo could influence the nature of N_2_ adsorption and activation, while the binding and desorption energies of N-containing intermediates are closely related with the d-band centers. Therefore, we investigated the d-band center of the RuCo DSAC surface by ultraviolet photoemission spectroscopy (UPS) and projected density of states (PDOS) calculation. The UPS spectrum (Fig. 4a) shows that RuCo DSAC has a d-band width narrower than that of bulk Co and Ru foil.^57^ Also, the d-band width of RuCo DSAC is obviously narrower than that of traditional RuCo alloy, as revealed in Fig. S15 (for more details see the ESI†). These results strongly suggest that the d bands of Co and Ru in RuCo DSAC are narrow, which is further implied by the results of PDOS calculation (Fig. 4b–e). For RuCo DSAC, the PDOS of Co over RuCo DSAC (Fig. 4c) significantly shifts towards the Fermi level in comparison with that of bulk Co (Fig. 4b), and the corresponding d-band center of Co shifts from −1.30 eV for bulk Co to −1.65 eV for RuCo DSAC (Table S5†). Also, the shift of the d-band center of Co is obviously more than that of Ru. In comparison with bulk Co (Fig. 4g), the PDOS (Fig. 4h) of RuCo DSAC shows that the e_g_ and t_2g_ states of Co in RuCo DSAC are almost degenerate, suggesting almost overlapping t_2g_ and e_g_ PDOS, and there is a nearly uniform coordination field for the Co 3d states. These results imply that the electronic structure of the Co 3d state in RuCo DSAC resembles that of a “free-Co atom”.^39^ It is expected that such unique electronic structure could promote desorption of N-containing intermediates, and thus have a beneficial influence on low-temperature NH_3_ synthesis.

### NH_3_ synthesis performance

**Fig. 5:**
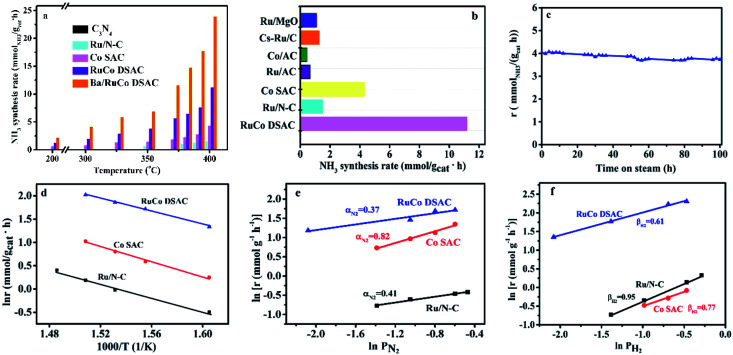
Catalytic performances. (a and b) NH_3_ synthesis performances: (a) NH_3_ synthesis rate at different temperatures and 1 MPa, and (b) NH_3_ synthesis rate of selected Ru and/or Co catalysts for NH_3_ synthesis at 400 °C and 1 MPa. (c) Time course of NH_3_ synthesis rate over the RuCo DSAC sample at 350 °C and 1 MPa. (d) Arrhenius plots. (e and f) Reaction orders: (e) N_2_ reaction order and (f) H_2_ reaction order at 400 °C and 1 MPa.

The catalytic performances of the C_3_N_4_ support and as-synthesized samples for NH_3_ synthesis were evaluated in a 25% N_2_–75% H_2_ feed at a WHSV of 60 000 mL g^−1^ h^−1^ as a function of reaction temperature. Obviously, there is only insignificant NH_3_ production over the C_3_N_4_ support within the test temperature range, which is less than 0.04 mmol_NH_3__ g_cat_^−1^ h^−1^ even at 400 °C. Meanwhile, the NH_3_ synthesis rates over the three samples in the range of 200–400 °C differ significantly (Fig. 5a). The addition of Ru to Co SAC significantly enhances the NH_3_ synthesis rate (Fig. 5a), and the rate of RuCo DSAC is much higher than that of the monometallic Ru and Co catalysts. For example, the NH_3_ synthesis rate over RuCo DSAC at 200 °C is 1.24 mmol_NH_3__ g_cat_^−1^ h^−1^, which is 8.2-fold that of monometallic Co SAC (0.15 mmol_NH_3__ g_cat_^−1^ h^−1^). However, there is hardly any activity over Ru/N–C below 350 °C. Also, the RuCo DSAC outperforms the Cs-promoted Ru/C catalyst, which is one of the most active NH_3_ synthesis catalysts, by 13.6 times at 200 °C (Table S6†). At 400 °C, the NH_3_ synthesis rate over RuCo DSAC is 11.20 mmol_NH_3__ g_cat_^−1^ h^−1^, which is 2.6-fold and 7.5-fold that of monometallic Co SAC (4.20 mmol_NH_3__ g_cat_^−1^ h^−1^) and monometallic Ru/N–C (1.49 mmol_NH_3__ g_cat_^−1^ h^−1^) catalysts, respectively. It is noteworthy that the outlet CH_4_ concentration over RuCo DSAC is negligibly low (Fig. S16†) upon the NH_3_ synthesis at 400 °C for 25 h under 1 MPa, suggesting that under the adopted conditions the RuCo DSAC is highly stable. Moreover, because the Brunauer–Emmett–Teller (BET) surface areas of Ru/N–C (88 m^2^ g^−1^) and RuCo DSAC (189 m^2^ g^−1^) as determined by N_2_ adsorption measurements at 77 K (Fig. S17†) are significantly different (Table S1†), we obtained the surface-area-normalized NH_3_ synthesis rates and made a comparison (Fig. S18†). An NH_3_ synthesis rate of 6.36 × 10^−6^ mmol m^−2^ s^−1^ was acquired at 350 °C on RuCo DSAC, while that over monometallic Ru/N–C is negligible. To find out whether the N species of the Ru–Co catalyst could be involved in NH_3_ synthesis, we exposed the Ru–Co catalyst to 75% H_2_/Ar at 400 °C and 1 MPa, and the cumulative amount of NH_3_ as a function of time is provided in Fig. S19.† It can be seen that the NH_3_ synthesis rate first increases to peak at 60 min, and then decreases sharply with time prolonging under a 75% H_2_/Ar atmosphere. The NH_3_ synthesis rate is lower than 0.1 mmol_NH_3__ g_cat_^−1^ h^−1^ after 250 min. To further confirm that the NH_3_ produced mainly originates from the catalytic synthesis of N_2_ gas rather than the nitrogen source of N-doped carbon support, the NH_3_ synthesis rate of RuCo DSAC has also been measured at 400 °C and 1 MPa using the feed gas of 75% H_2_–25% 15N_2_, instead of 25% N_2_–75% H_2_. Our studies show that the NH_3_ synthesis rate in the presence of 75% H_2_–25% 15N_2_ (10.59 mmol_NH_3__ g_cat_^−1^ h^−1^) is slightly lower than that of 75% H_2_–25% N_2_ (11.20 mmol_NH_3__ g_cat_^−1^ h^−1^) at 400 °C and 1 MPa (Fig. S20†), showing that the NH_3_ produced mainly originates from the catalytic synthesis of N_2_–H_2_ mixed gases. The H_2_-TPD-MS experiment was also carried out (Fig. S21†). The signal of *m*/*z* = 17 is very close to the baseline, showing that the production of ammonia can be ignored. However, a very weak signal of *m*/*z* = 32 can be discerned, indicating that there are only tiny amounts of dynamic N in the catalyst that can be reacted with hydrogen.

In addition, the NH_3_ synthesis rate over RuCo DSAC at 400 °C exhibits an approximately linear increase from 11.20 to 20.39 mmol_NH_3__ g_cat_^−1^ h^−1^ when the pressure is raised from 1.0 to 5.0 MPa (Fig. S22†). The catalytic performance of RuCo DSAC at such pressure range should permit convenient eHB operation on a large scale. To further reveal the unique intrinsic catalytic activity, we calculated the turnover frequency (TOF). It is well known that Ru and Co entities are both active for NH_3_ synthesis, and therefore it is difficult to differentiate the catalytic contribution of the two. Herein, TOF_M_ was calculated (more details are provided in the Experimental section) to express catalytic activity on a per-M-active-site basis. Interestingly, TOF_Ru_ and TOF_Co_ over RuCo DSAC reach 0.016 s^−1^ and 6.7 × 10^−3^ s^−1^ (Fig. S23†), respectively, at 400 °C, which is 10.7-fold that of monometallic Ru/N–C and 5.8-fold that of Co SAC. Therefore, it is reasonable to attribute the effective formation of NH_3_ under mild conditions to strong synergism between the dual atomically dispersed Ru and Co active centers.

Previous studies have shown that the addition of a proper promoter to Ru or Co-based catalysts could promote NH_3_ synthesis.^58,59^ In the present work, we studied the effect of adding Ba into the best-performing RuCo DSAC (5 wt% against RuCo DSAC) on the NH_3_ production rate. As displayed in Fig. 5a, the results reveal that the NH_3_ synthesis rate of Ba/RuCo DSAC is (1.8–3)-fold that of the non-promoted one, depending on the reaction temperature. Surprisingly, when the temperature is 400 °C, the NH_3_ synthesis rate over Ba/RuCo DSAC is 23.90 mmol_NH_3__ g_cat_^−1^ h^−1^. Moreover, we compared the NH_3_ synthesis rates of Ba/RuCo DSAC (Table S6†) and RuCo DSAC (Fig. 5b) with those of selected Ru- and Co-based catalysts, and they are superior to those of the conventional Ru- and Co-based catalysts.

The long-term stability test of RuCo DSAC was conducted at 350 °C for 100 h, and its NH_3_ synthesis rate remains almost constant after a slight decrease at the initial stage (Fig. 5c). The used RuCo DSAC was subject to TEM, HR-TEM, AC-STEM and XRD analyses. The TEM (Fig. S24a and b†) and HR-TEM images (Fig. S24c and d†) of the used RuCo DSAC show no Ru or Co NPs, and the AC-STEM images (Fig. S25a and b†) of the used RuCo DSAC still show a large number of individual bright dots, revealing the retention of the Ru and Co atomic dispersion even after a stability test of 100 h. The XRD patterns (Fig. S26†) of the used Ba/RuCo DSAC and RuCo DSAC samples indicate the absence of Ru or Co phases. One characteristic peak that is related to the g-C_3_N_4_ phase can still be observed after the NH_3_ synthesis reaction, suggesting high thermal stability of g-C_3_N_4_. Meanwhile, the H_2_-TPR (Fig. S27†) profile of the C_3_N_4_ support shows no reduction peak below 550 °C. Also, the H_2_-TPR profile of RuCo DSAC exhibits no reduction peaks below 500 °C. In addition, the Ar-TPD-MS analysis of the as-synthesized fresh catalysts does not show any desorption or decomposition peak below 600 °C (Fig. S28†). These results undoubtedly confirm that the C_3_N_4_ support and RuCo DSAC are stable under the reaction for NH_3_ synthesis. Evidently, the RuCo DSAC is not only active but also stable under the adopted mild conditions for NH_3_ synthesis.

### Kinetic studies

The kinetic parameters of NH_3_ synthesis such as activation energies and reaction orders over the as-synthesized catalysts are depicted in Fig. 5d–f. The apparent activation energy (*E*_a_) of NH_3_ synthesis over the as-synthesized catalysts was derived from the Arrhenius plots depicted in Fig. 5d. The *E*_a_ is determined to be 58 kJ mol^−1^ for RuCo DSAC, which is lower than that of Ru/N–C (79 kJ mol^−1^) or Co SAC (65 kJ mol^−1^). The *E*_a_ value of RuCo DSAC is similar to that of previously reported Ru-loaded electrides (50–60 kJ mol^−1^)^11,26,60^ and hydrides (49–60 kJ mol^−1^).^6,25^ Moreover, experiments to determine the reaction orders of N_2_ and H_2_ over the as-prepared catalysts were performed. For traditional Ru-based catalysts in NH_3_ synthesis, the N_2_ reaction order is between 0.8 and 1.0 while that of H_2_ is negative in value. The former suggests that N_2_ dissociation is the rate-limiting step, while the latter indicates the poisoning effect of H_2_ on Ru.^61,62^ It was observed that the reaction orders of N_2_ (Fig. 5e), H_2_ (Fig. 5f) and NH_3_ (Fig. S29†) over RuCo DSAC are 0.37, 0.61 and −1.07, respectively. The results of the positive H_2_ reaction order indicate that the effect of H_2_ poisoning on the Ru sites is circumvented over RuCo DSAC. The reaction orders for the RuCo DSAC are different from those for Ru/N–C and Co SAC, consistent with the presence of the dual single-atom active centers that may create the cooperativity that favors N_2_ adsorption/activation and H_2_ dissociative adsorption occurring separately on different sites. In this regard, we performed surface-science characterization and theoretical modeling to acquire insights into the roles of the dual single-atom active centers at the atomic level for NH_3_ synthesis.

## Discussion

A suite of elaborate characterization and DFT calculations were employed to explore the adsorption and activation behavior of N_2_ and H_2_. In H_2_-TPD (Fig. S30a†) and N_2_-TPD (Fig. S30b†) studies, the desorption temperatures of H_2_ and N_2_ over RuCo DSAC and monometallic Ru/N–C as well as Co SAC are slightly different, but they are all below 200 °C, suggesting that the adsorption and activation of H_2_ and N_2_ are not difficult on these catalysts. For Ru/N–C and Co SAC, there is detection of mass signals of *m*/*z* = 32 (Fig. S31a†) and *m*/*z* = 17 (Fig. S31b†), respectively, corresponding to the desorption of *N_2_H_4_ and *NH_3_ species accumulated on the catalyst surface during NH_3_ synthesis, and desorption of these species can still be observed even at 700 °C. It is to be noted that the signal intensity of *m*/*z* = 32 and *m*/*z* = 17 over the used RuCo DSAC is much lower, the former getting close to the baseline at *ca.* 200 °C, while the latter can be ignored even at 50 °C. This observation is in good agreement with the UV-vis DRS experiment (for more details see the ESI†) and DFT calculation reported below, displaying that the energies needed for *N_2_H_4_ and *NH_3_ desorption over RuCo DSAC are only 0.2 eV and 0.1 eV, respectively. These results strongly suggest facile desorption of surface *N_2_H_4_ and *NH_3_ intermediates from RuCo DSAC. Moreover, an *in situ* DRIFTS deuterium labeling investigation was performed to determine the reactivity of intermediate species for NH_3_ synthesis. For the fresh C_3_N_4_ sample, there is detection of bands at 1637, 1406, 1316, and 1236 cm^−1^ which are characteristic of the CN heterocycle.^63^ After C_3_N_4_ was exposed to a mixture of 25% N_2_–75% D_2_ or 25% N_2_–75% H_2_ at 400 °C for 30 min, there is no detection of any additional IR peaks in comparison with fresh C_3_N_4_ (Fig. S32†). The observation is in accord with the fact that C_3_N_4_ shows almost no catalytic activity. After Ru/N–C and Co SAC were exposed to a mixture of 25% N_2_–75% D_2_ at 400 °C, there is detection of bands at 2394 cm^−1^ that are related to the v_5_(B_u_)N_2_D_2_ transition or N–D torsion modes within the ND_2_ or N_2_D_4_ fragments,^64–68^ and the band intensity significantly increases with prolonged exposure from 1 to 30 min, further indicating the accumulation of *N_2_D_*x*_ species on the surface of Ru/N–C (Fig. S31c†) and Co SAC (Fig. S31d†). These results indicate that the surface Ru or Co active sites are partially covered by the intermediate species, which can only desorb at high temperature (>400 °C). Interestingly, such a troublesome scenario is virtually eliminated in the case of RuCo DSAC (Fig. S33†). Specifically, the band at 2394 cm^−1^ is attributed to the v_5_(B_u_)N_2_D_2_ transition or N–D torsion modes within the ND_2_ or N_2_D_4_ fragments. Two bands located at 2574 cm^−1^ and 1545 cm^−1^ can also be discerned, which can be attributed to *trans*-HNND and NN stretching in N_2_D_*x*_ species, respectively.^66^ The formation of *trans*-HNND may be a result of NND interaction with the surface H left behind in the pretreatment of the catalyst in 10% H_2_/Ar at 400 °C for 2 h. These results further confirm the existence of N_2_D_*x*_ species. The UV-vis DRS spectra (Fig. S34†) show that the main intermediate species of RuCo DSAC in NH_3_ synthesis is N_2_H_4_, and the peak intensity of the N_2_H_4_ compound decreases with the increase of reaction temperature.

From these observations and analyses, it follows that *N_2_H_4_ is likely the main intermediates under the reaction conditions, and the presence of *N_2_H_4_ also suggests that the activation of N_2_ is more facile *via* hydrogenation to NNH than *via* direct dissociation of N≡N triple bonds. Moreover, the accumulation of species such as *N_2_H_4_ and *NH_3_ on Ru/N–C and Co SAC will block the sites for further activation of N_2_ and H_2_, thus hindering NH_3_ formation at low temperature over both samples. For RuCo DSAC, the immediate implications of the involvement of the dual single-atom active sites with narrow d-band centers are the interruption of the scaling correlation between the adsorption/activation of N_2_ and desorption of surface N-containing species, and hence the superior low-temperature NH_3_ synthesis performance upon the decoupling of scaling correlation.

**Fig. 6:**
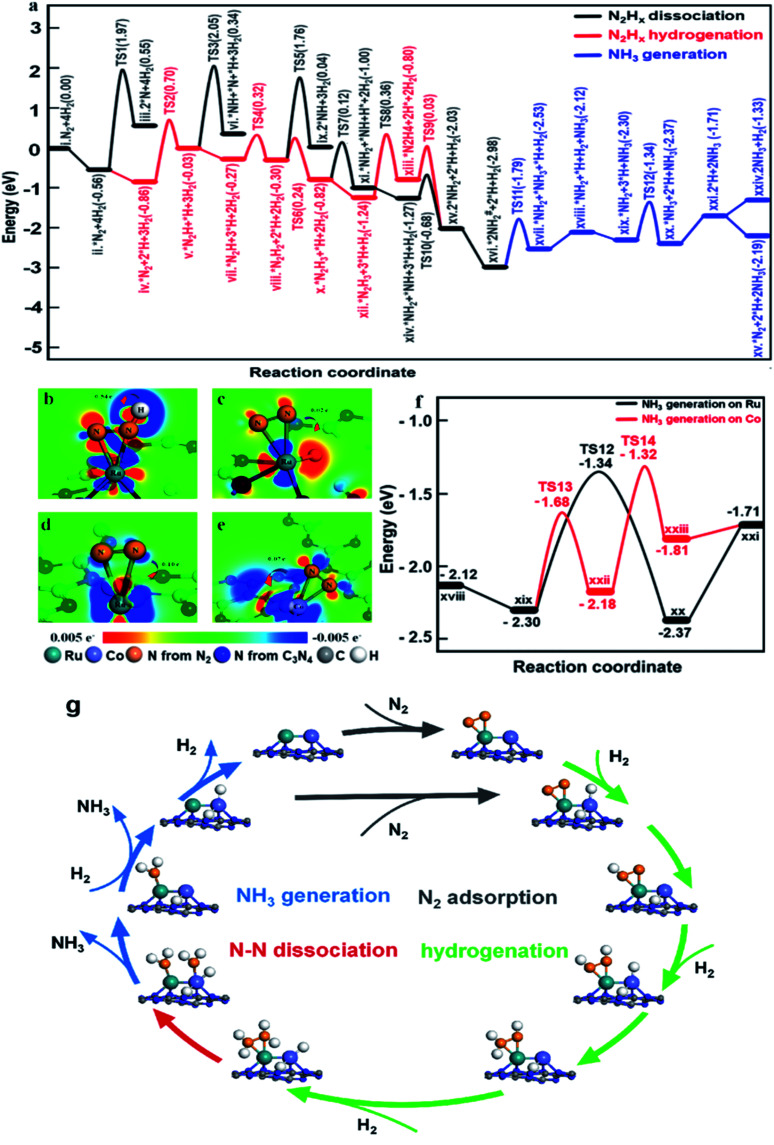
DFT calculations of NH_3_ synthesis on the RuCo DSAC. (a) Reaction pathway on the RuCo DSAC. (b–e) Charge density differences over nitrogen adsorbed on (b and c) Ru–Co DSAC, (d) Ru SAC and (e) Co SAC sites with the adsorption of H_2_. The red area shows an increase of electron density while the blue area indicates a decrease. (f) NH_3_ generation on different adsorption sites of RuCo DSAC. (g) Schematic of the NH_3_ synthesis reaction pathway over RuCo DSAC.

To illustrate the role of dual single-atom active centers and to deduce the possible reaction pathway over RuCo DSAC, DFT calculations were performed and the calculation results are shown in Fig. 6a–e and S35–S39.† From these results, the Ru atom is the dominant site for N_2_ adsorption, due to the higher adsorption energy (−0.56 eV, Fig. S36a†) on Ru than on the Co atom (−0.43 eV, Fig. S36c†). Notably, the adsorption of a N_2_ molecule on a single Ru atom tends to adopt the side-on coordination (Fig. S36a and c†), due to symmetry matching between the π* orbitals of N_2_ and 3d orbitals of Ru. Then, the adsorbed N_2_ on the Ru site is more facile to be hydrogenated than direct N_2_ dissociation. The energy for directly breaking the N≡N bond under the active state on the Ru atom is still up to 2.53 eV, indicating difficult direct dissociation. In general, the cleavage of N≡N triple bond needs at least two adjacent Ru atoms.^69,70^ The absence of Ru–Ru ensembles as revealed in EXAFS and AC-STEM analyses over RuCo DSAC implies that the direct dissociation of N_2_ is unlikely, which is also consistent with the determination of *N_2_H_4_ desorption in Ar-TPD measurements (Fig. S31a†). Then, the N_2_ molecule on the Ru site needs to be activated before the occurrence of hydrogenation (Fig. 6a). The Co site as a bridge accelerates the electron transfer from g-C_3_N_4_ to the Ru site, which promotes the donation of 3d electrons from the Ru atom to the π* orbitals of N_2_ and weakening of N≡N bonds as mentioned earlier.^69^

On the other hand, the dissociative adsorption of H_2_ over the Co active center of RuCo DSAC is efficient (Fig. S37a–d†), where H_2_ adsorption is thermodynamically exothermic throughout the entire reaction pathway. Also, the Co atom plays an important role in gathering and activating H_2_ for N_2_ hydrogenation at the Ru atom. The above analysis indicates that the adsorption and dissociation of N_2_ and that of H_2_ occur at different active sites. The significant advantage of RuCo DSAC is that the competitive adsorption of N_2_ and H_2_ can be effectively avoided, as reflected by the positive reaction order of H_2_ (Fig. 5f). Comparing the charge density differences (Fig. 6c–e) of N_2_ and H_2_ co-adsorption on RuCo DSAC (Fig. 6c) and Co or Ru SAC (Fig. 6d and e) sites, electron filling of π* orbitals decreases with the drop of electron density on N_2_, clearly showing that the existence of H_2_ poses a negative effect on N_2_ activation, and it is more obvious in the latter (charge of N_2_ shift more than 0.7 e^−^). H_2_-TPD results (Fig. S30a†) show that the amount of H_2_ desorption from RuCo DSAC (1.40 mmol g^−1^) is much higher than the Co coverage, therefore it is reasonable to deduce that there is a spillover of H atoms from the Co atom to Ru active centers.

What follows is the attack of activated N_2_ on Ru by spillover H atoms to generate N_2_H_*x*_ species. Note that when the N–N bond becomes weak enough the cleavage of the N–N bond and the generation of *NH_2_ species may take place. Fig. 6a displays the energy profiles of the full pathway for NH_3_ production (from state i to xxi), and the pathways of N–N bond dissociation (states iii, iv, ix, and xi) and hydrogenation (from state ii to xiii) for comparison. The first hydrogenation step for the generation of *N_2_H (from state ii to iii) needs to overcome an energy barrier of 1.56 eV, much lower than that of direct N_2_ breakage (2.53 eV). Upon the formation of *N_2_H, the N=N bonds are further weakened as indicated by the charge density differences shown in Fig. 6b. Both the hydrogenation of *N_2_H species and the cleavage of the N–N bond of *N_2_H_*x*_ species are much easier than the direct dissociation of N_2_, demonstrating the unique function of H atoms for N_2_ activation (Fig. 6a). Notably, the entire pathway of N_2_ hydrogenation is in the presence of excessive adsorbed H atoms, and the Ru site with spillover H atoms can keep the adsorption of N_2_ activated throughout as illustrated in Fig. 6a.

Subsequently, the *N_2_H species is further hydrogenated to *N_2_H_2_, *N_2_H_3_ and *N_2_H_4_ intermediates with energy barriers in the range of 0.11–1.20 eV. With the stepwise hydrogenation of *N_2_H_*x*_ species, the energy barrier for breaking the N–N bond keeps falling (2.53 eV for *N_2_, 2.08 eV for *N_2_H, 2.06 eV for *N_2_H_2_, 0.94 eV for *N_2_H_3_ and 0.83 eV for *N_2_H_4_), showing that N_2_ hydrogenation is an effective way to sharply weaken the N–N bond. Finally, the indirect cleavage of the *H_2_N–NH_2_ bond takes place together with the formation of two *NH_2_ species. It is noteworthy that the activation energy for *N_2_H_3_ dissociation is also lower than that for *N_2_H_3_ hydrogenation, thus the cleavage of the N–N bond of *N_2_H_3_ is also possible for the generation of the *NH_2_ intermediate. Then, the transfer of the *NH_2_ species from the Ru atom to the Co atom is favorable because of the crowding at the Ru site. With active H atoms available on the Co site, further hydrogenation of *NH_2_ to *NH_3_ on the Co site is ready to occur. Compared to the Ru site, the Co site is more favorable for NH_3_ desorption, releasing the first NH_3_ molecule to the gas phase. It is remarkable that the reaction energies for the generation of the second NH_3_ on the Co sites and Ru sites are similar (−1.32 eV and −1.34 eV, Fig. 6f), while the desorption energy of *NH_3_ on the Co atom (0.10 eV) is much lower than that on the Ru atom (0.66 eV), indicating that NH_3_ desorption from the Co sites is more preferred in comparison to that from the Ru site.

Overall, two significant findings have been acquired in the present study. First, with the Ru and Co dual single-atom active site, a new route for NH_3_ synthesis is made possible (Fig. 6g), which is essentially different from those of previously reported monometallic Co or Ru catalysts as well as traditional alloy clusters.^71,72^ According to the results of experimental and theoretical investigations, electron release and/or donation between Ru and Co atoms could promote N_2_ adsorption and activation as well as hydrogenation, while the narrow d-band centers of Ru and Co allow desorption of surface intermediate species such as *N_2_H_4_ and *NH_3_ at low temperature. Therefore, the superior performance of RuCo DSAC in NH_3_ synthesis under mild conditions is attributed to the cooperativity of dual single-atom Ru and Co centers. Second, NH_3_ synthesis over RuCo DSAC proceeds *via* the associative pathway, similar to that occurring in the cases of metal single atoms,^24,57^ clusters,^23,36^ Li-promoted Ru catalysts and Co_3_Mo_3_N.^73,74^ Over the RuCo DSAC in N_2_ activation to NH_3_, the step with the highest kinetic barrier is the hydrogenation of N_2_ to generate *N_2_H on the Ru site, which is the rate-determining step. After considering the entropy contribution, the entropy contribution in the RDS step is only 0.007 eV. Notably, it was experimentally observed that *N_2_H_4_ is the major detected species, which can be ascribed to the fact that the stepwise hydrogenation of *N_2_H to *N_2_H_2_, *N_2_H_3_ and *N_2_H_4_ has much lower energy barriers than that of the “*N_2_ + H → *N_2_H” or “*N_2_H_4_ → *2NH_2_” process. The net outcome is a steady state of *N_2_H_4_ presence and hence easy detection of N_2_H_4_.

## Conclusions

To summarize, we have successfully synthesized a RuCo DSAC, in which there is integration of Ru atoms onto an atomically dispersed cobalt surface in the form of RuCo dual single-atom sites on g-C_3_N_4_. We found that the RuCo DSAC structure could effectively facilitate electron transfer from pyrrolic N to the surface Ru atom, which acts as an efficient site for N_2_ adsorption and activation as well as hydrogenation. The RuCo DSAC with narrow d-band centers favors desorption of surface intermediate species such as *N_2_H_4_ and *NH_3_ at low temperature. The cooperativity of Ru and Co centers decouples the scaling relation by providing separated sites discretely for N_2_ activation and *N_2_H/*NH_3_ desorption, respectively. The NH_3_ synthesis rate over the RuCo DSAC is about 2.6–8.2-fold that of monometallic Co at 200–400 °C and 6.2–7.5-fold that of Ru at 350−400 °C. It is anticipated that the cooperative roles of Ru and Co disclosed in the present study shed light on the design of dual single-atom active sites that could enable energy-efficient NH_3_ synthesis under mild conditions.

## Experimental section

### Chemicals and materials

Melamine and ruthenium nitrosyl nitrate solution were purchased from Shanghai Aladdin Biochemical Technology Co., Ltd. Cobalt phthalocyanine (CoPc) and cyanuric acid were from Shanghai Macklin Biochemical Co., Ltd. Dimethyl sulfoxide (DMSO) and ethanol were from Sinopharm Chemical Reagent Co., Ltd. High purity argon (99.9999%) and nitrogen (99.9999%) gases were supplied by Linde Industrial Gases. The N_2_–H_2_, H_2_–Ar and CO–He mixed gases of designated proportions were also from Linde Industrial Gases. D_2_ (99.999%) was purchased from Cambridge Isotope Laboratories, Inc.

### Catalyst preparation

#### Preparation of RuCo DSAC

Typically, 0.35 mL of ruthenium nitrosyl nitrate solution, 0.201 g of CoPc and 0.50 g of melamine were mixed and dissolved in 40 mL of DMSO under ultrasonic treatment for 10 min to obtain a blue solution. Meanwhile, 0.51 g of cyanuric acid was dissolved in 10 mL of DMSO under ultrasonic treatment for 10 min to give a transparent solution. Then, the transparent solution was slowly added into the blue solution and the resulting mixture was stirred at room temperature for 10 min. After filtration and washing with 150 mL deionized water and 100 mL ethanol, the solid precursor was obtained. Finally, the obtained precursor was dried at 60 °C for 12 h, followed by thermal polymerization at 600 °C under an Ar atmosphere for 8 h in a tube furnace at a ramp rate of 1 °C min^−1^.

The synthetic procedure of Ru/N–C and Co/N–C SAC was similar to that of RuCo DSAC, except for the absence of CoPc and ruthenium nitrosyl nitrate solution, respectively.

For the Ba-promoted RuCo DSAC, 5 wt% Ba(NO_3_)_2_ was added to the RuCo DSAC *via* an incipient wetness impregnation (IWI) method.

### NH_3_ synthesis performance

Before the evaluation of catalytic performance for NH_3_ synthesis, the samples (0.15 g, diluted with quartz powder in a 1 : 8 volumetric ratio) were reduced in a flow of 25% N_2_–75% H_2_ at 400 °C for 4 h. Under the conditions for NH_3_ synthesis in a 25% N_2_–75% H_2_ mixture at a WHSV of 60 000 mL g^−1^ h^−1^ and a given pressure, the outlet NH_3_ concentrations were measured using a known amount of diluted H_2_SO_4_ solution (1 mol L^−1^) and analyzed by ion chromatography (Thermo Scientific, DIONEX, ICS-600). Finally, the NH_3_ synthesis rates were acquired based on the outlet NH_3_ concentrations. Turnover frequency (TOF_M_, M = Co or Ru) was acquired by dividing the NH_3_ synthesis rate by the total number of Co or Ru atoms.

### Experiments for methanation determination

For the determination of the possibility of methanation, 0.2 g of RuCo DSAC was exposed to a flow of 25% N_2_–75% H_2_ at 400 °C at a WHSV of 60 000 mL g^−1^ h^−1^ under a pressure of 1 MPa. The outlet CH_4_ concentration was detected using an online GC-mass spectrometer (GCMS-QP2010 SE).

### Measurement of N_2_ and H_2_ reaction orders

The sample (0.25 g) was first exposed to a mixture of 10% H_2_/Ar at 350 °C for 4 h before the introduction of N_2_ or H_2_. All measurements were conducted at 350 °C at a designated pressure, and the outlet NH_3_ was measured using a known amount of diluted H_2_SO_4_ solution (0.02 mol L^−1^) and analyzed by ion chromatography (Thermo Scientific, DIONEX, ICS-600). Finally, the NH_3_ synthesis rates based on the outlet NH_3_ concentrations were calculated. The constituent gases in volume fraction of the reactant feed (N_2_, H_2_, Ar) were as follows in volume fraction (12.5%, 37.5%, 50%), (35%, 37.5%, 27.5%), (45%, 37.5%, 17.5%) and (55%, 37.5%, 7.5%) for the N_2_ reaction order and (25%, 62.5%, 12.5%), (25%, 50%, 25%), (25%, 25%, 50%) and (25%, 12.5%, 62.5%) for the H_2_ reaction order.

### Materials characterization

Powder X-ray diffraction (XRD) was performed (at 40 kV and 40 mA) on a Panalytical X'Pert Pro diffractometer using Cu-Kα radiation (*λ* = 0.1789 nm). The Brunauer–Emmett–Teller (BET) surface area and Barrett–Joyner–Halenda (BJH) pore size distribution were measured by N_2_ adsorption–desorption on a Micromeritics ASAP 2020 instrument at −196 °C after the sample was degassed at 120 °C for 2 h in a vacuum. Inductively coupled plasma atomic emission spectroscopy (ICP-AES) analysis was conducted using an Ultima 2 spectrometer. Scanning electron microscopy (SEM) was performed on a Hitachi Model S-4800 microscope operated at 5 kV. Transmission electron microscopy (TEM) and high-resolution transmission electron microscopy (HR-TEM) were conducted on a JEM-2010 microscope.

Aberration-corrected high-angle annular dark-field scanning transmission electron microscopy (HAADF-STEM) was conducted on a JEOL JEM-ARM 200 F instrument equipped with a CEOS probe corrector, with a guaranteed resolution of 0.08 nm.

### Ultraviolet photoelectron spectroscopy (UPS)

UPS measurements were conducted using a helium resonance lamp which provided He I (*hν* = 21.2 eV) and He II (*hν* = 40.8 eV) photons (1 eV = 0.16 aJ).

### H_2_ temperature-programmed reduction (H_2_-TPR)

A H_2_-TPR experiment was performed on an AutoChem II 2920 equipped with a thermal conductivity detector (TCD), in which samples were first pretreated under Ar flow (30 mL min^−1^) at 400 °C for 0.5 h. After cooling to room temperature, the temperature was increased from RT to 800 °C at 5 °C min^−1^ in a gas flow of 10 vol% H_2_/Ar (30 mL min^−1^).

### H_2_ temperature-programmed desorption (H_2_-TPD-MS) experiment

First, the fresh RuCo DSAC was pretreated with H_2_ at 400 °C for 2 h and then cooled to room temperature, and then temperature-programmed desorption was performed. In this process, the signals of *m*/*z* = 32 and 17 were collected using the mass spectrometer.

### NEXAFS measurements

The N K-edge and Co L-edge near-edge X-ray absorption fine structure spectroscopy (NEXAFS) measurements were performed at the BL12B beamline of the Beijing Synchrotron Radiation Facility. The bending magnet was connected to the beamline, which is equipped with three gratings covering photon energies from 100 to 1000 eV with an energy resolution of *ca.* 0.2 eV. The NEXAFS signal was detected at room temperature using the surface-sensitive total electron yield (TEY) mode by recording the sample drain current. The resolving power of the grating was typically *E*/Δ*E* = 1000, and the photon flux was 1 × 10^−10^ photons per second.

### XANES and EXAFS measurements

X-ray absorption near-edge structure (XANES) and extended X-ray absorption fine structure (EXAFS) analyses were conducted at the 1W2B beamline of the Beijing Synchrotron Radiation Facility. Before the test, the sample was firstly treated with 25% N_2_–75% H_2_ at 400 °C for 2 h. The Co and Ru K-edge spectra of the samples and reference samples in transmission mode were measured at room temperature. A Si(111) double-crystal monochromator was used to abate the harmonic content of the monochromatic beam.

### XPS measurements and Ar^+^ etching

X-ray photoelectron spectroscopy (XPS) measurements were performed on an ESCALAB 250Xi photoelectron spectrometer (Thermo Fisher Scientific) equipped with a monochromatic Al-Kα source (*K*_α_ = 1486.6 eV) and a charge neutralizer. The XPS binding energy was calibrated against the C 1s peak at 284.6 eV of adventitious carbon. Prior to *in situ* measurements, XPS spectra of the fresh sample were acquired. Argon ion etching was carried out with the MAGCIS dual mode ion source, which can be operated as a monatomic argon ion source, and the monatomic mode at an energy of 1000 eV was selected.

### *In situ* DRIFTS deuterium labeling experiments

An *in situ* DRIFTS D_2_-isotopic labeling experiment was performed using a Nicolet Nexus FT-IR spectrometer. The sample was first reduced in a 10% H_2_/Ar mixture at 400 °C for 4 h. After collecting the background spectrum, the catalyst was exposed to a 25% N_2_–75% D_2_ mixture at 400 °C for different periods.

### Temperature-programmed Ar desorption

An Ar-TPD-MS experiment was conducted by mass spectrometry using an Autochem 2920 instrument. After the activity test of NH_3_ synthesis, 50 mg of the used catalyst was flushed with Ar before being heated to 700 °C at a rate of 10 °C min ^−1^. The *m*/*z* = 32 (N_2_H_4_) and 17 (NH_3_) signals during desorption were recorded.

### UV-vis absorption spectra

A UV-vis DRS experiment was performed using a PerkinElmer Lambda 750s UV-visible spectrometer. We installed a flask trap containing sulfuric acid solution and *para*-(dimethylamino)benzaldehyde at the exit of the reactor during the NH_3_ synthesis reaction. The collected solution was then used for the UV-vis measurements.

### Electron-paramagnetic resonance (EPR) measurements

EPR measurements were carried out on an E500 spectrometer (Bruker-BioSpin) with a 100 kHz magnetic field in the X band at RT.

### Computational method

First-principles calculations based on spin-polarized density functional theory (DFT) were performed using the Vienna *Ab initio* Simulation Package (VASP)^75^ and the projected augmented wave (PAW) method.^76^ The generalized gradient approximation with the Perdew–Burke–Ernzerhof (PBE) exchange–correlation functional was employed.^77^ The kinetic energy cutoff in plane-wave expansion was set as 400 eV with 2 × 2 × 1 Monkhorst–Pack grids involved in the Brillouin zone integration.^78^ The convergence thresholds of the energy change and the maximum force for the structural optimizations were set as 10^−5^ eV and 0.02 eV Å^−1^, respectively. The energies of species were corrected with zero-point energies and the thermodynamic data of gas-phase molecules were obtained from NIST (http://cccbdb.nist.gov/). The catalyst models were constructed on C_3_N_4_ with loaded metal atoms, adopting a vacuum space thickness of 15 Å. Four different structures were built and optimized as depicted in the ESI.† The energy of Co@C_3_N_4_ and Ru@C_3_N_4_ SACs was set as the reference and the most stable structure with the lowest energy was used for further calculations.

## Conflicts of interest

The authors declare no competing financial interest.

## Supplementary Material

SC-012-D1SC00304F-s001
