# Supplementary material for: Highly efficient ammonia synthesis at low temperature over a Ru–Co catalyst with dual atomically dispersed active centers
Source: Chem Sci. 2021 Apr 7;12(20):7125–37. doi: 10.1039/d1sc00304f (PMC8153211; doi:10.1039/d1sc00304f)
Supplement: SC-012-D1SC00304F-s001 [file SC-012-D1SC00304F-s001.pdf]

Electronic Supplementary Material (ESI) for Chemical Science.
This journal is © The Royal Society of Chemistry 2021

Supplementary Information

for

Highly efficient ammonia synthesis at low temperature over Ru-Co dual single-atom catalyst

Xuanbei Peng[a,†], Han-Xuan Liu[b,†], Yangyu Zhang[a,†], Zheng-Qing Huang[b], Linlin Yang[a], Yafei Jiang[c], Xiuyun Wang[a]*, Lirong Zheng[d], Chunran Chang[b], Chak-tong Au[a], Lilong Jiang[a]* and Jun Li[c,e]

[a]National Engineering Research Center of Chemical Fertilizer Catalyst, Fuzhou University, Fuzhou, Fujian, 350002, China.

[b]Shaanxi Key Laboratory of Energy Chemical Process Intensification, School of Chemical Engineering and Technology, Xi'an Jiaotong University, Xi'an 710049, China.

[c]Department of Chemistry, Southern University of Science and Technology, Shenzhen, China.

[d]Institute of High Energy Physics, Chinese Academy of Sciences, Beijing, China.

[e]Department of Chemistry, Tsinghua University, Beijing, China.

†These authors contributed equally.

*E-mail: xywang2017@fzu.edu.cn; jllfzu@sina.cn/jll@fzu.edu.cn.

Supplementary methods.

Synthesis of reference samples

The Ru/C material was prepared via incipient wetness impregnation. Briefly, commercial C was impregnated with a stoichiometric amount of ruthenium nitrosyl nitrate solution to obtain a Ru loading of 1.34 wt%, then dried at 60 °C for 12 h, and finally calcined at 500 °C for 3 h. The synthetic procedure of Co/C catalyst was similar to that of Ru/C, except that ruthenium nitrosyl nitrate was replaced by CoPc, and the Co loading amount was 2.24 wt.%. For the Cs-Ru/C catalyst, commercial C was impregnated with a designated amount of $CsNO_3$ and ruthenium nitrosyl nitrate solution to acquire a loading of 5 wt% Cs and 1.34 wt% Ru.

For the Ru/MgO catalyst, MgO was prepared by precipitation method. Briefly, magnesium nitrate was dissolved in deionized water. Sodium carbonate solution was then dropwise added with vigorous stirring. The resultant slurry was stirred for 0.5 h and washed several times with deionized water until pH=7, then aged at 40 °C for 4 h and dried at 120 °C for 24 h, followed by calcination at 500 °C for 3 h.

Results

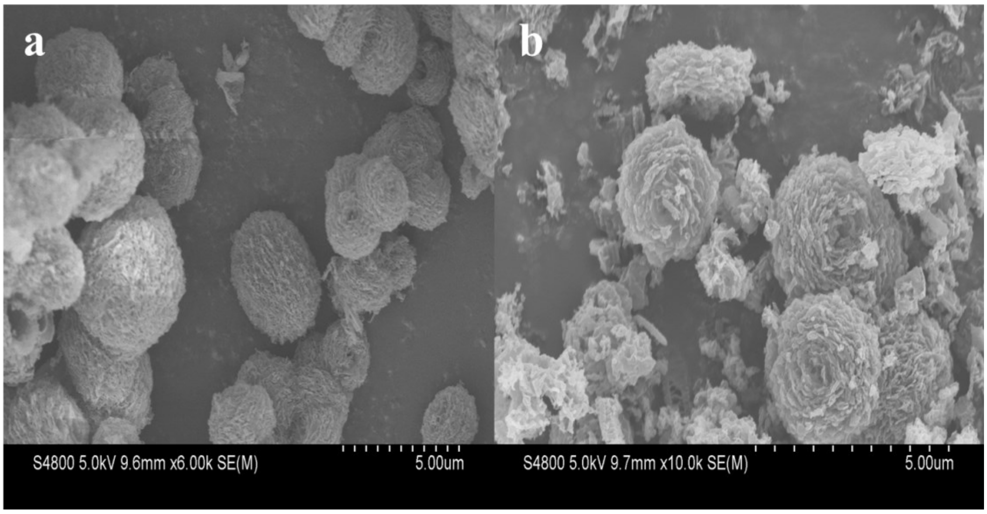

Fig. S1 SEM images of **a.** Ru/N-C and **b.** Co SAC catalyst.

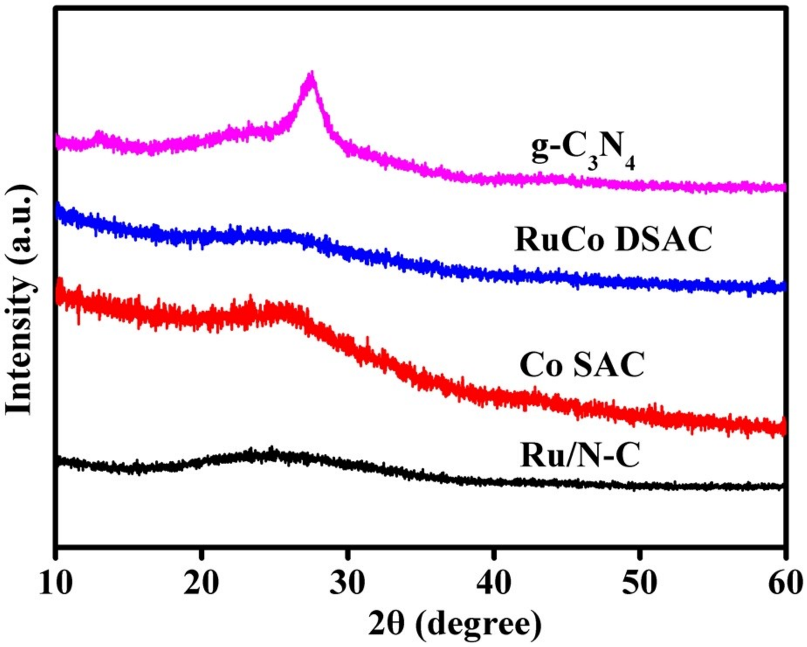

Fig. S2 XRD pattern of as-prepared catalysts.

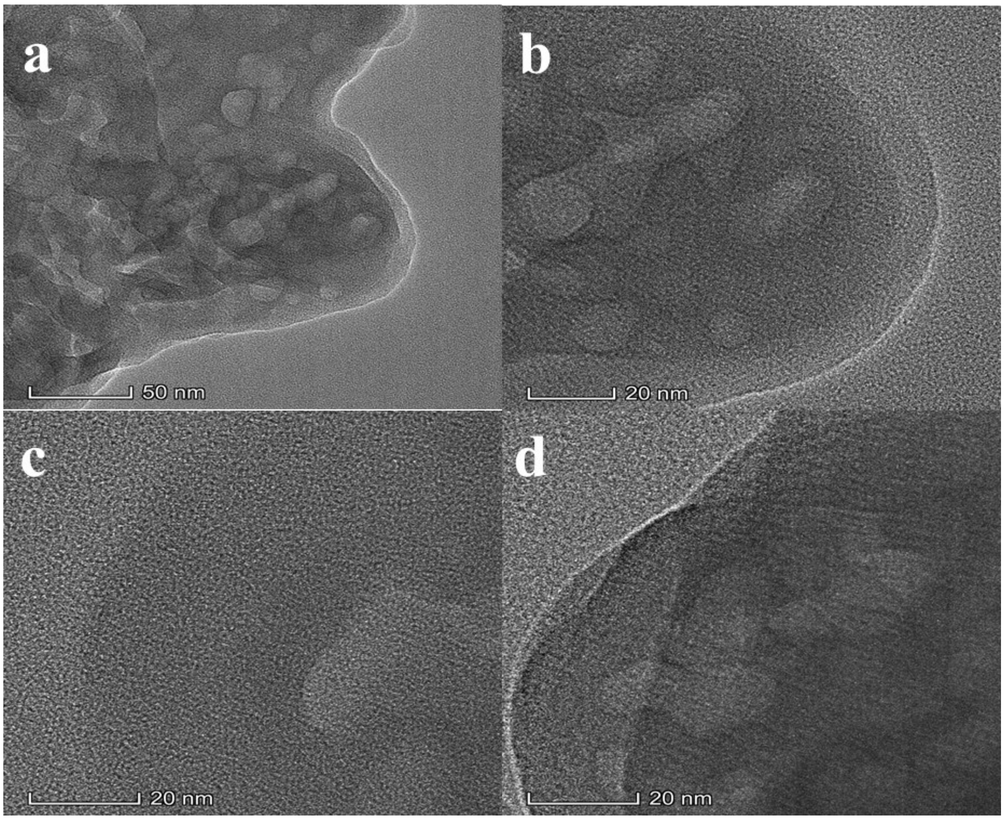

Fig. S3 a-d. TEM images of RuCo DSAC catalyst at different selected areas.

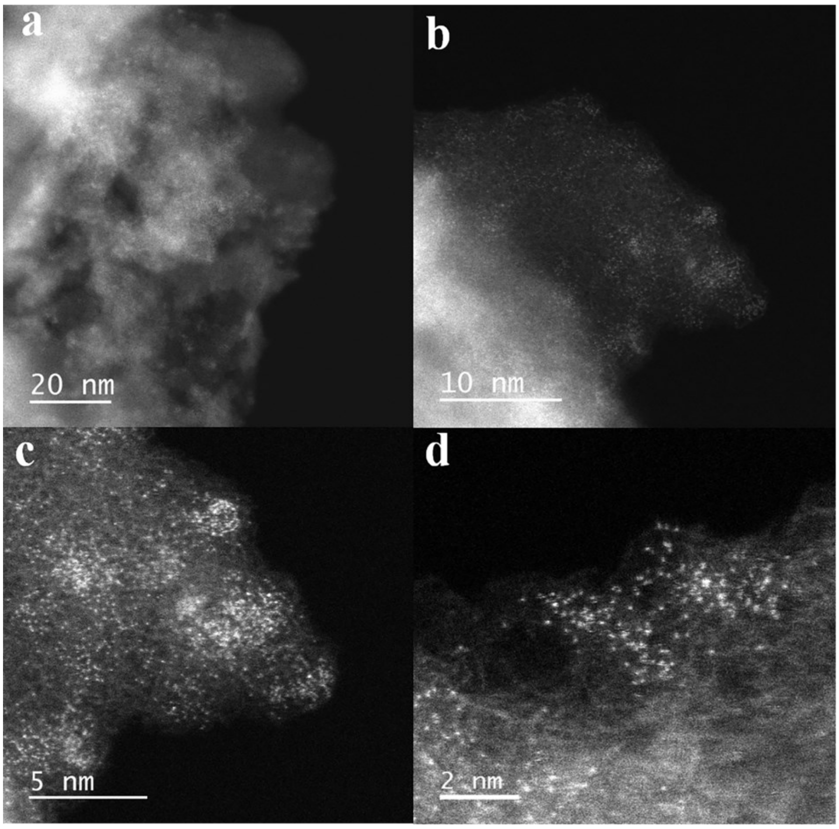

Fig. S4 a-d. Aberration-corrected STEM images distribution over of Ru/N-C catalyst.

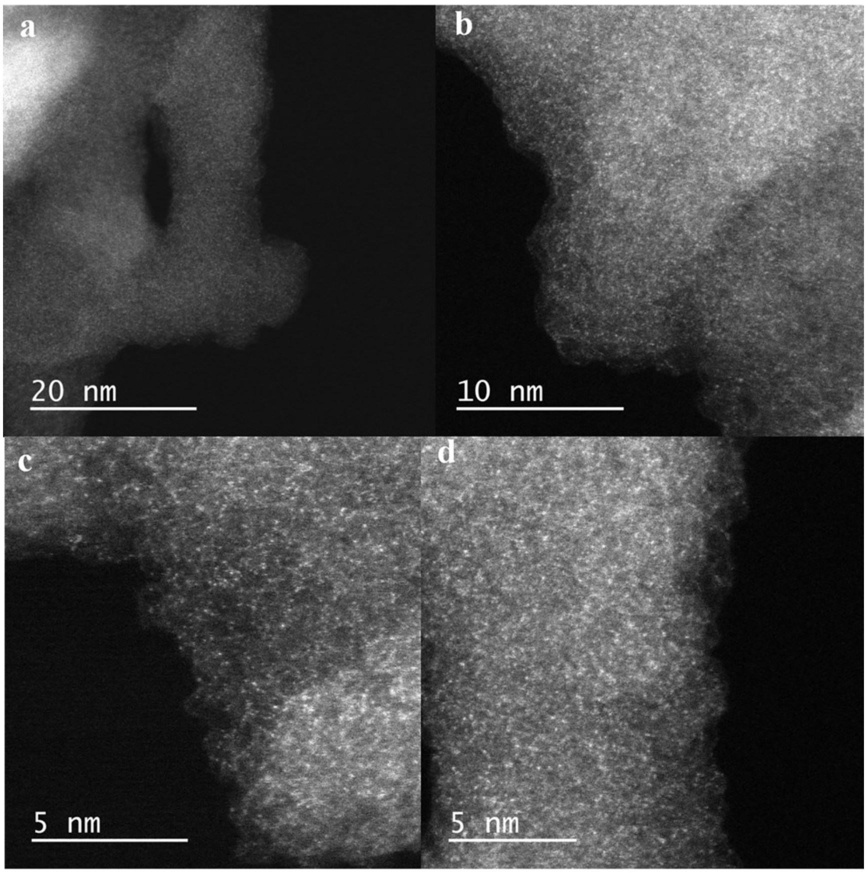

Fig. S5 a-d. aberration-corrected STEM images of Co SAC catalyst.

The aberration-corrected STEM images of Co SAC show many individual bright dots (Figure S5a-d). They represent the presence of Co atoms which are much heavier than that of C and N atoms. The individual Co atoms uniformly dispersed throughout the Co SAC catalyst. Single Co atoms are repeatedly observed in different regions of the catalyst, further revealing that Co predominantly exists as single atoms rather than in the form of small clusters or nanoparticles.

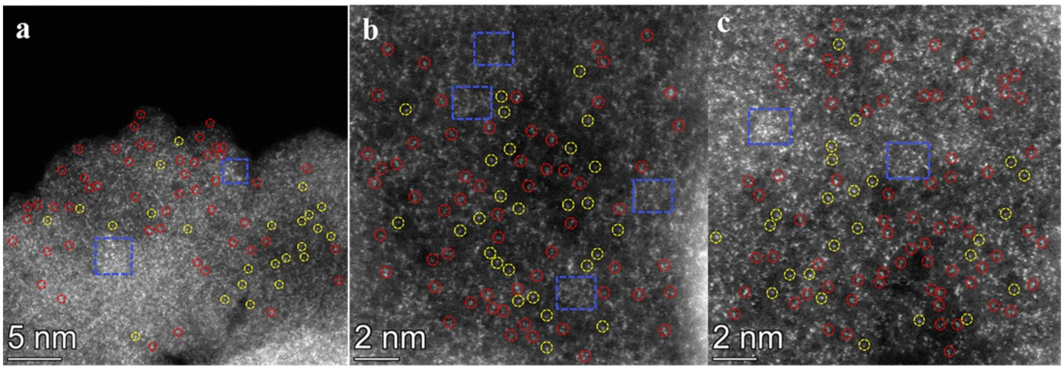

Fig. S6 a-c. Aberration-corrected STEM images of RuCo DSAC catalyst taken at different magnifications.

The red dotted circles, yellow dotted circles as well as blue dotted squares marked in Fig. S6 represent one single Ru atom adjacent to a Co atom, individual Ru or Co atoms and one single Ru atom adjacent with several Co, respectively.

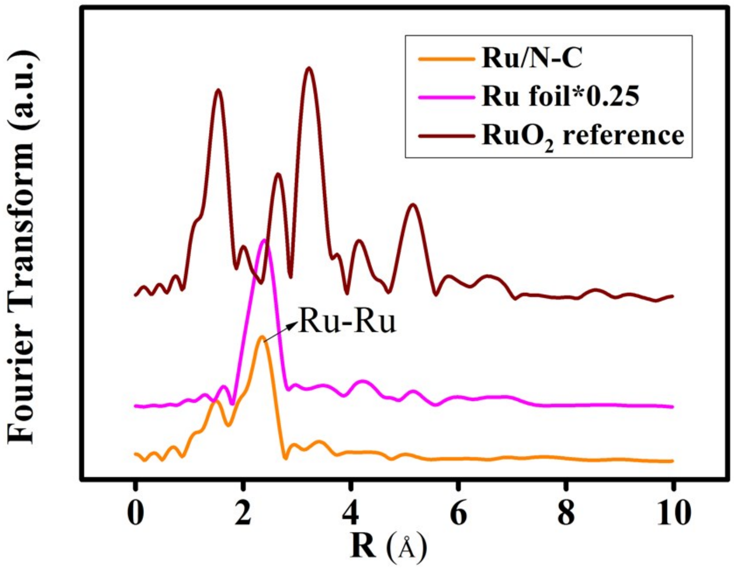

Fig. S7 Ru K-edge EXAFS spectra of Ru/N-C and reference samples.

A pronounced peak at ca. 2.3 Å is observed over Ru/N-C, matching well with that of metallic Ru-Ru, indicating aggregation of Ru atoms.

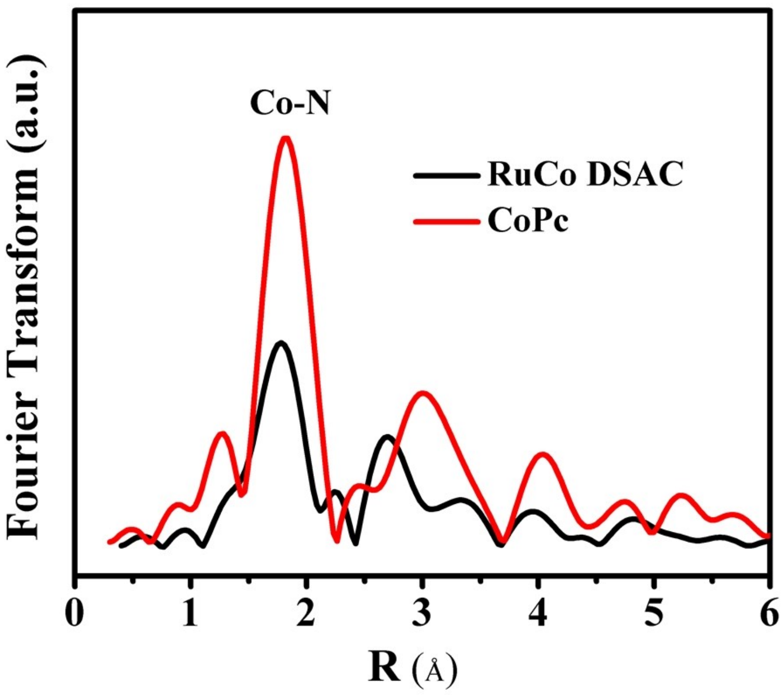

Fig. S8 Co K-edge EXAFS spectra of CoPc and RuCo DSAC.

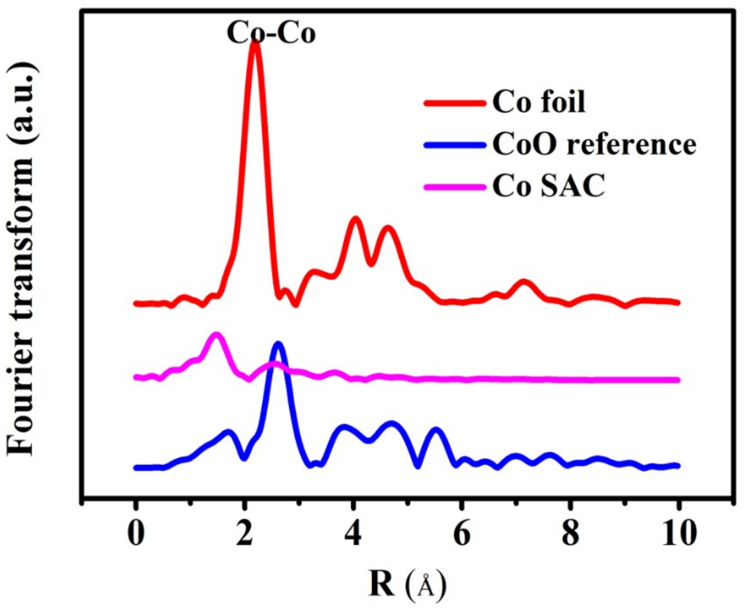

Fig. S9 Co K-edge EXAFS spectra over Co SAC, Co foil and CoO reference samples.

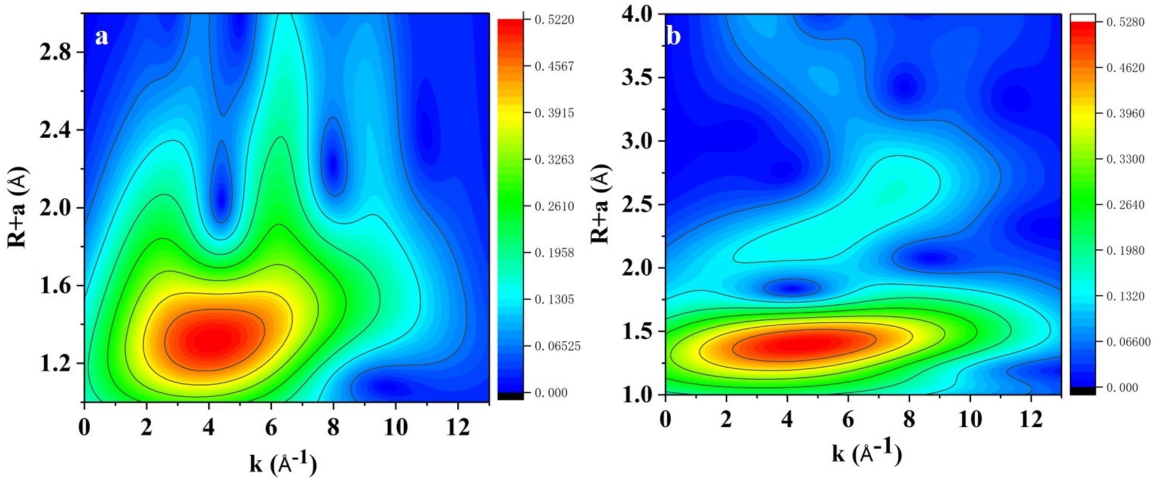

Fig. S10 a-b. Wavelet transform plots on the **a.** Ru K-edge, **b.** Co K-edge EXAFS signal over RuCo DSAC.

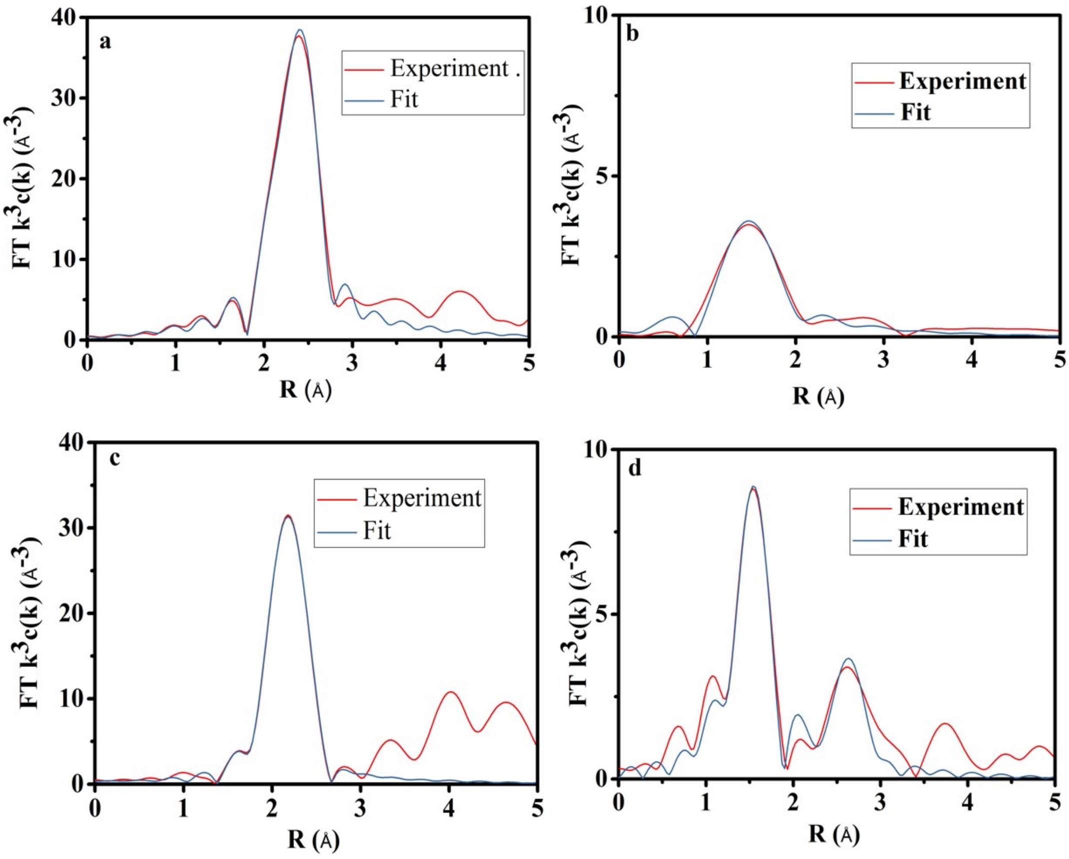

Fig. S11 a-d. EXAFS fitting results. **a.** Ru foil. **b.** Ru K-edge over RuCo DSAC**. c.** Co foil. **d.** Co K-edge over RuCo DSAC.

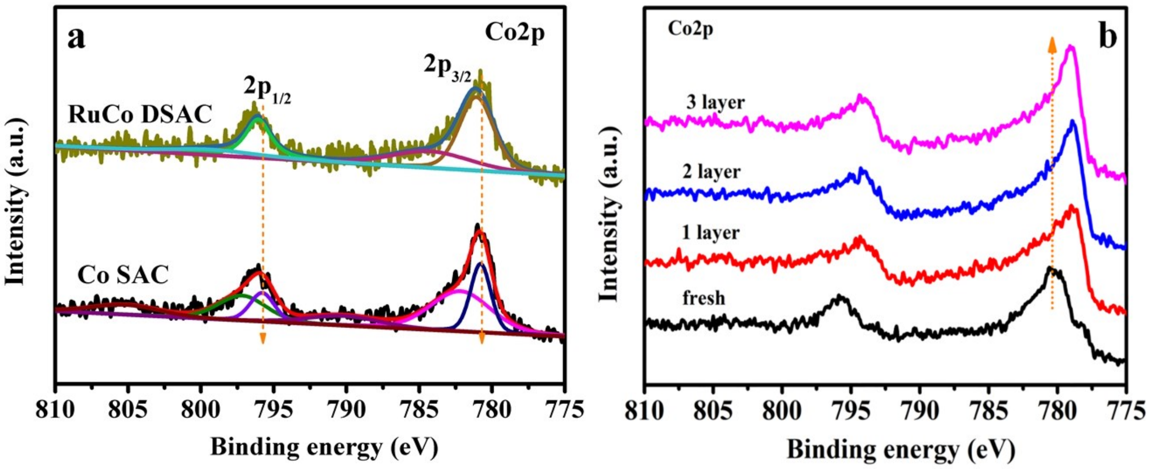

Fig. S12 a. XPS Co2p spectra of fresh samples, **b**. RuCo DSAC subject to Ar^+ etching with the number of layers removed indicated.

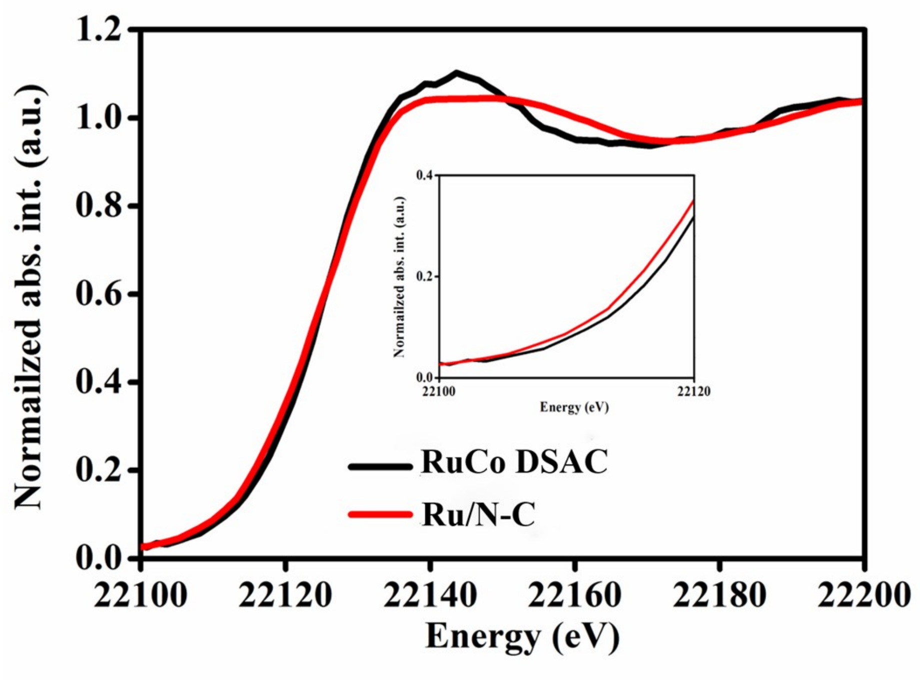

Fig. S13 Normalized Ru K-edge XANES spectra over Ru/N-C and RuCo DSAC catalysts.

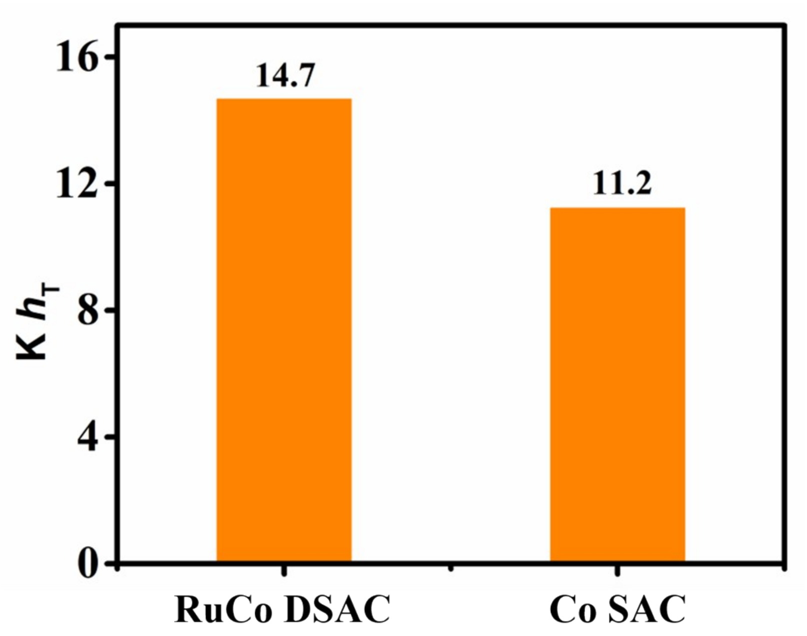

Fig. S14 Unoccupied Co3*d* charge (Kh_T) over RuCo DSAC and Co SAC catalysts acquired from Co L-edge NEXAFS spectra.

The transfer of Co 3*d* electrons and the number of d holes (i.e., unoccupied *d* electron states), were semiquantitatively calculated using the method proposed by Mattheiss and Dietz.[1] Briefly, the areas A_2 and A_3 under the L_2 and L_3 white lines are related to the number of *d* holes $h_{3/2}$ and $h_{5/2}$, respectively, with the following equation:[2]

$$A_2 = KR_{2p_{1/2}}(\frac{1}{3}h_{3/2}) \quad (1)$$

$$A_3 = KR_{2p_{3/2}}(\frac{1}{15}h_{3/2} + \frac{2}{5}h_{5/2}) \quad (2)$$

Where, $R_{2p1/2}$ and $R_{2p3/2}$ are the radial transition matrix elements; K is a constant characteristic of element. Assuming that $R_{2p1/2}$ is approximately equal to $R_{2p3/2}$, and marked as R_{2p}, the total areas A_T can be written as

$$A_T = A_2 + A_3 = KR_{2p}\frac{2}{5}(h_{2/3} + h_{5/2}) = KR_{2p}\frac{2}{5}(h_T) \quad (3)$$

In case of Co, R is 0.155.[3] Therefore, the total numbers of d holes h_T is given by

$$Kh_T = 16.13A_T \quad (4)$$

Since the constant K is not determined, we semiquantitatively estimated the Kh_T value of catalysts by comparing the numbers of *d* holes.

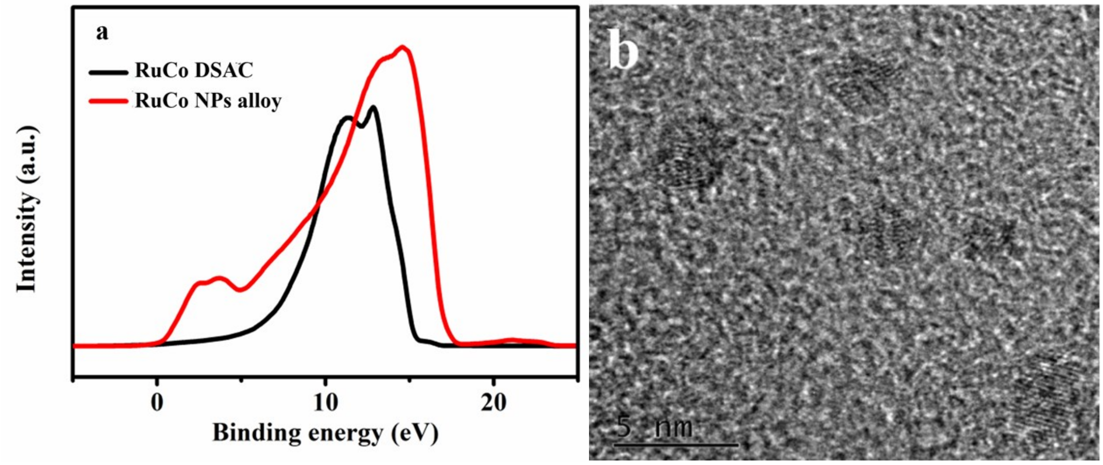

Fig. S15 UPS profiles of RuCo DSAC and RuCo nanoclusters alloy (Notes: The synthetic procedure of RuCo nanoclusters alloy was similar to that of RuCo DSAC, except for the molar ratio of Ru/Co. The molar ratio in the RuCo nanoclusters alloy catalyst is 1:1, and the images of RuCo nanoclusters alloy was confirmed by HR-TEM techniques.

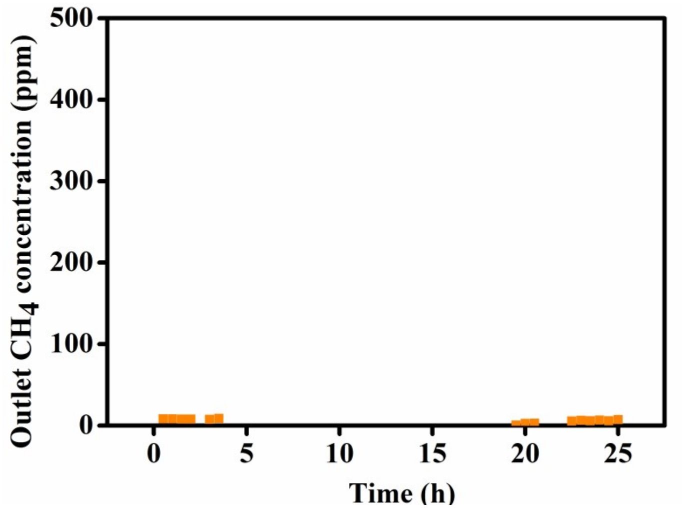

Fig. S16 The outlet CH_4 concentration as a function of time during NH_3 synthesis over RuCo DSAC sample at 400 °C (test conditions: WHSV= 60 000 ml g^{-1} h^{-1}, 1MPa).

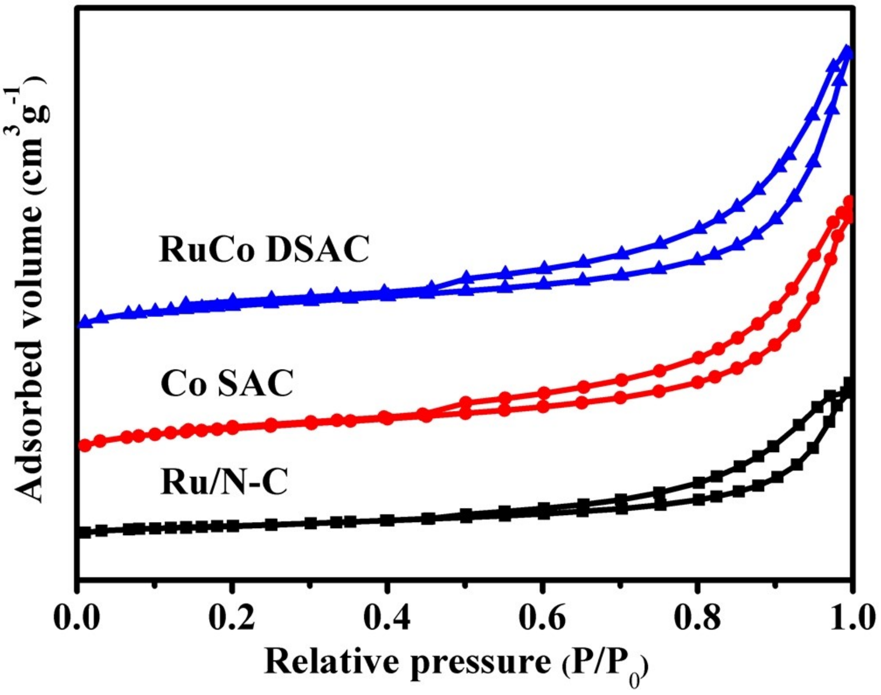

Fig. S17 N_2 adsorption-desorption isotherm of as-prepared catalysts.

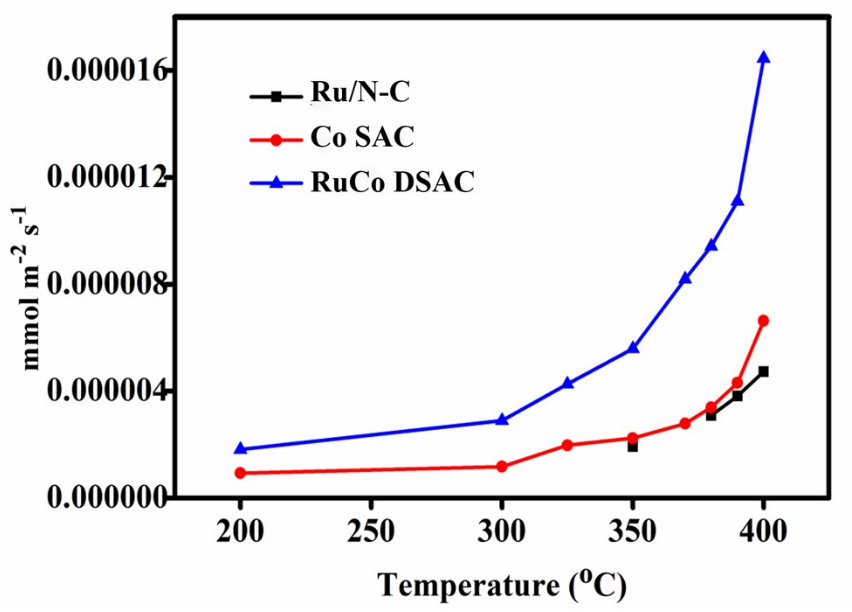

Fig. S18 Surface-area-normalized NH_3 synthesis rates of Co SAC, Ru/N-C and RuCo DSAC at 3 MPa versus reaction temperatures.

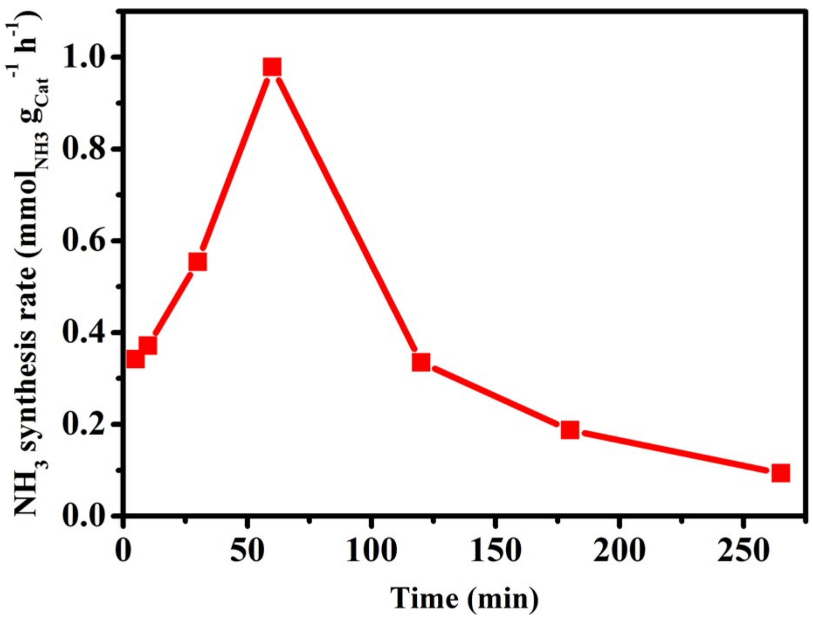

Figure S19. NH_3 synthesis rate as a function of time over RuCo DSAC in the presence of 75%H_2/Ar at 400 °C and 1 MPa.

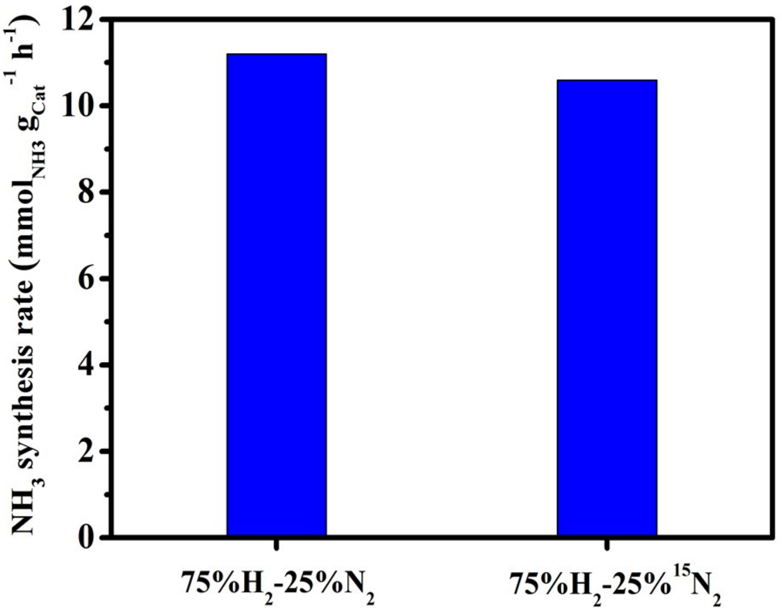

Figure S20. NH_3 synthesis rate over RuCo DSAC in the presence of 75%H_2-25%N_2 and 75%H_2-25%$^{15}N_2$ (Test conditions: 400 °C, 1 MPa and WHSV=60,000 mL g^{-1} h^{-1}).

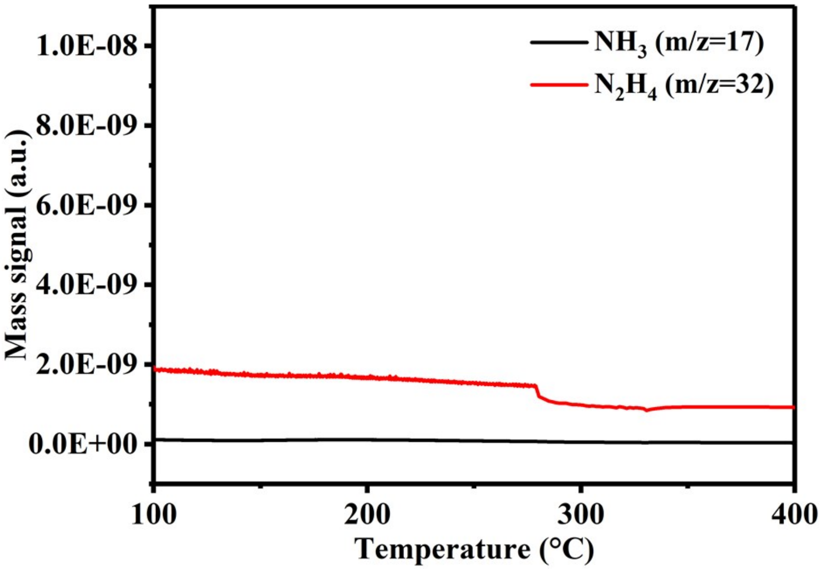

Fig. S21 TPD-MS profiles over RuCo DSAC catalyst.

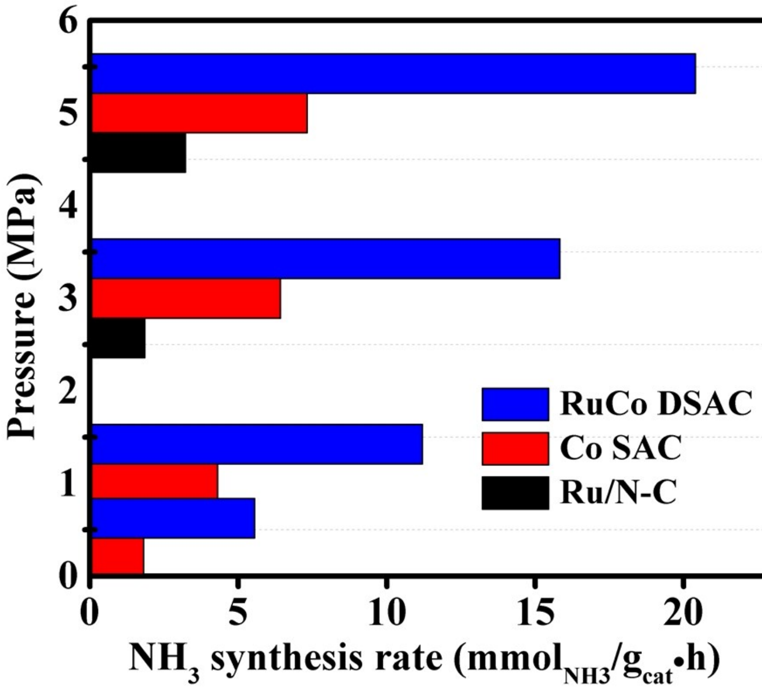

Fig. S22 NH_3 synthesis rates of as-synthesized at 400 °C with different pressures.

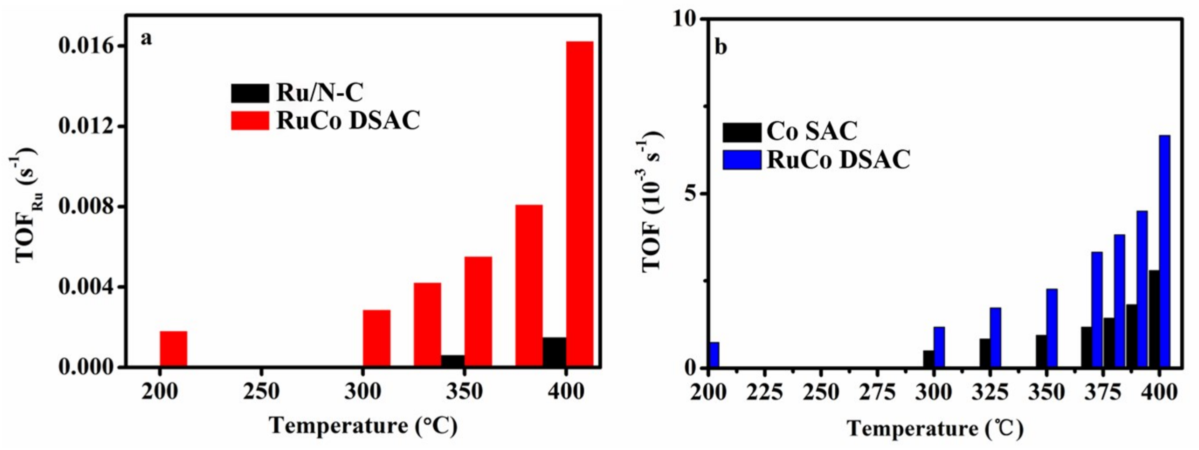

Fig. S23 Turnover frequencies (TOF_{Ru} and TOF_{Co}) at 1 MPa and designated temperatures: **a.** TOF_{Ru} and **b.** TOF_{Co} over as-prepared catalyst.

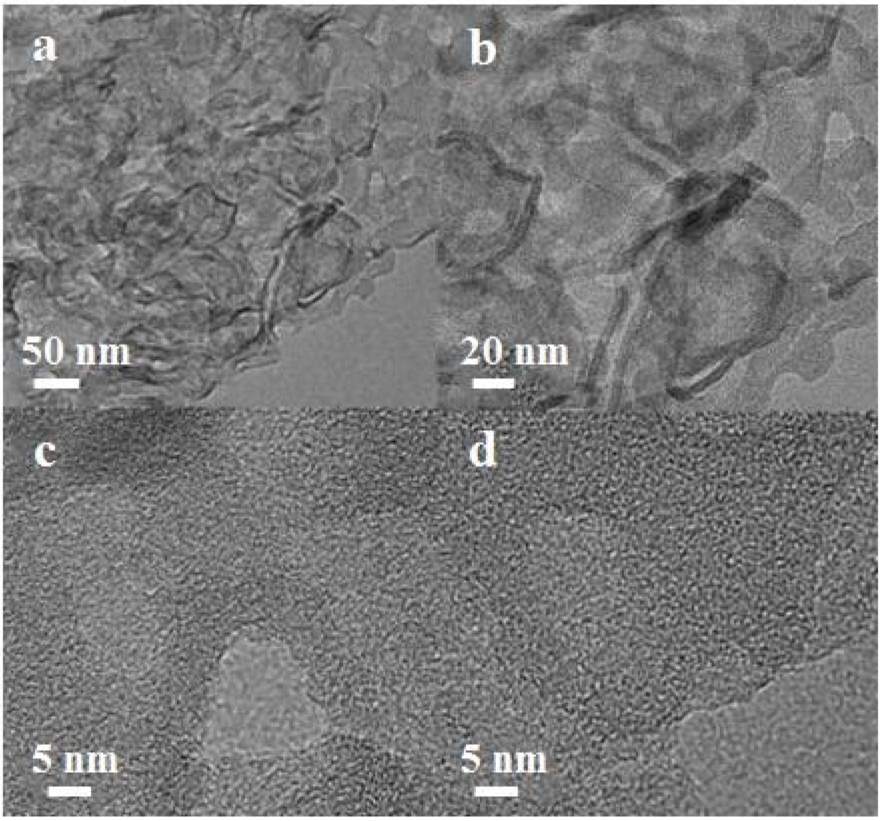

Fig. S24 a-b. TEM and **c-d**. HR-TEM images of the used RuCo DSAC catalyst after NH_3 synthesis reaction.

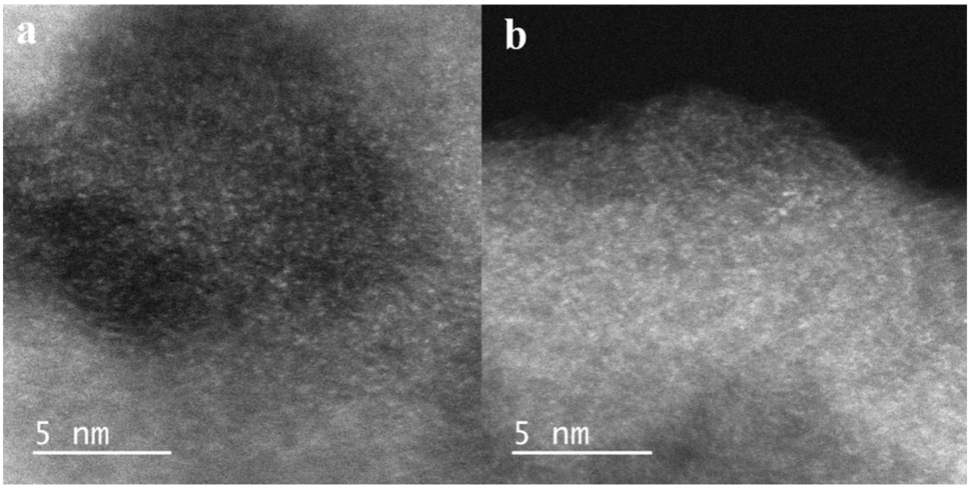

Fig. S25 a-b. Aberration-corrected high-angle annular dark field-scanning transmission electron microscopy images of RuCo DSAC after NH_3 synthesis reaction.

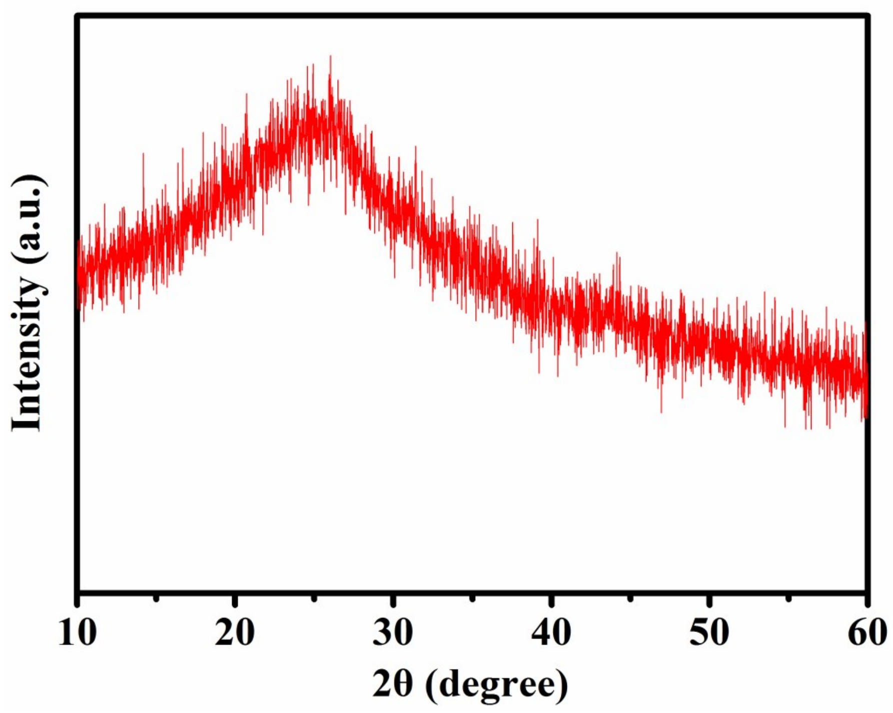

Fig. S26 XRD pattern of RuCo DSAC after NH_3 synthesis reaction.

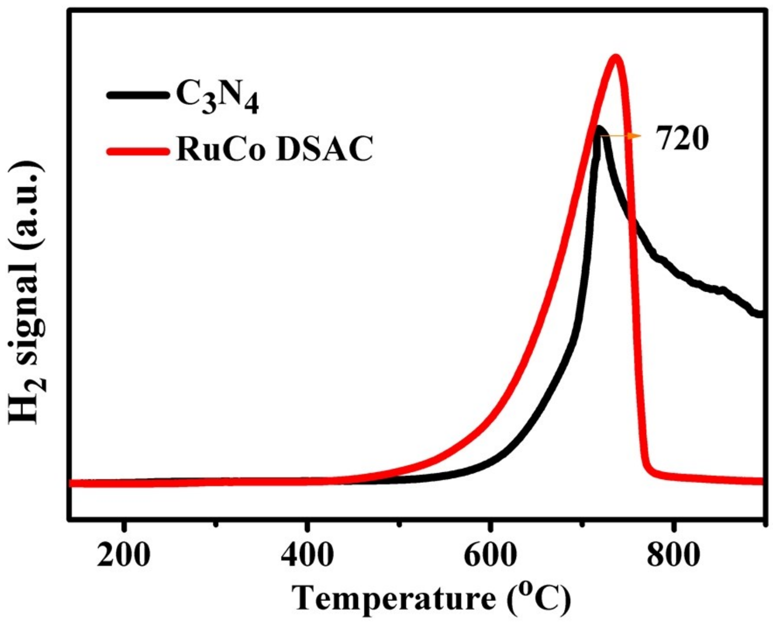

Fig. S27 H_2-TPR profile of C_3N_4 support and RuCo DSAC.

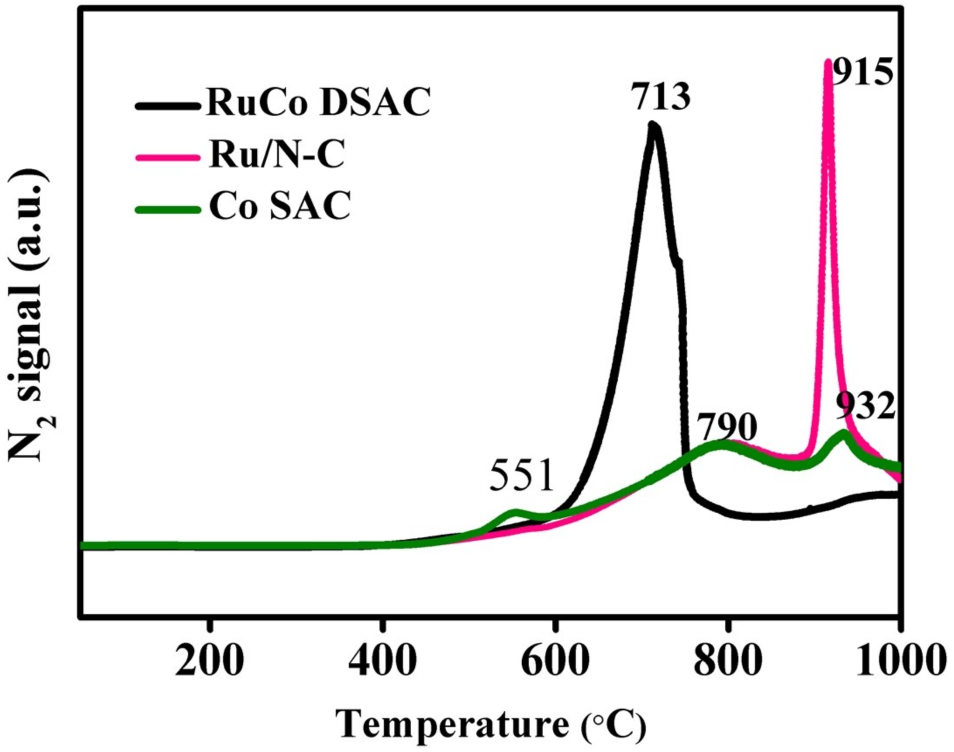

Fig. S28 Ar-TPD profiles of the as-synthesized fresh catalysts.

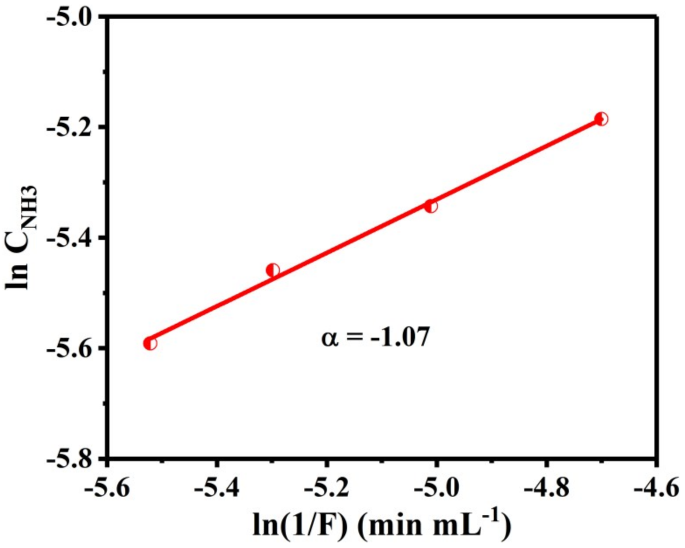

Fig. S29 Reaction order of NH_3 over RuCo DSAC.

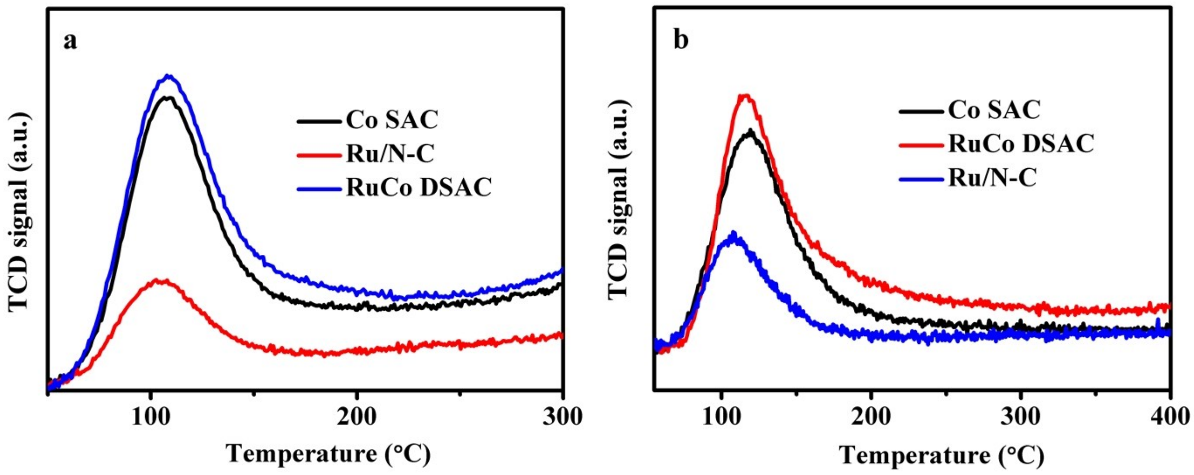

Fig. S30 **a**.H_2-TPD and **b.** N_2-TPD profiles over as-synthesized catalysts.

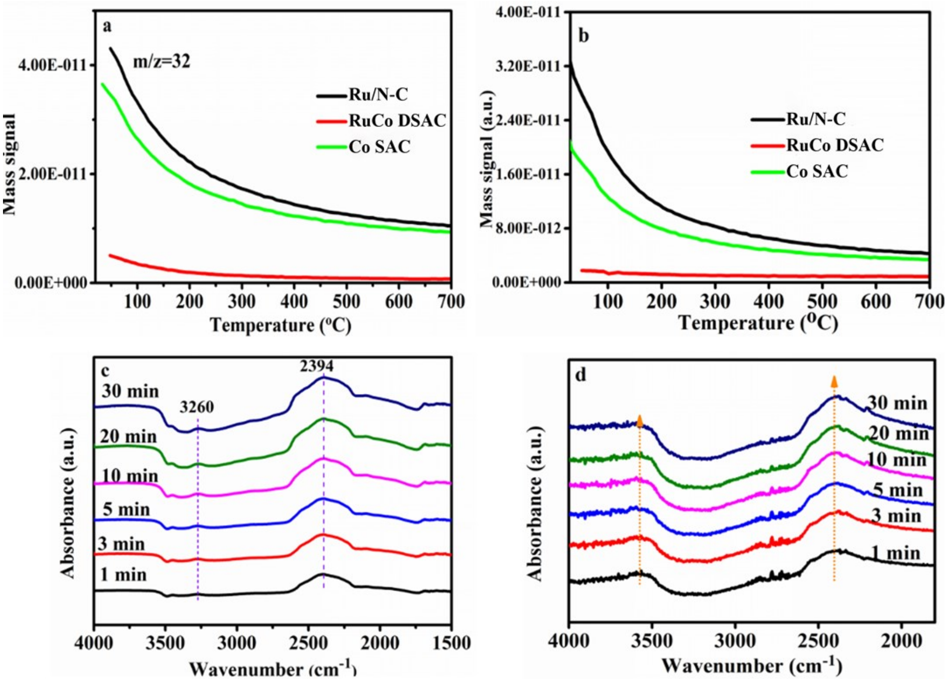

Fig. S31 a. m/z=32 and **b.** m/z=17 during Ar-TPD-MS experiments over as-synthesized catalysts. **Deuterium labeling**: *in situ* DRIFTS experiments of **c.** Ru/N-C and **d.** Co SAC samples after exposure to 25%N_2-75%D_2 mixture at 400 °C.

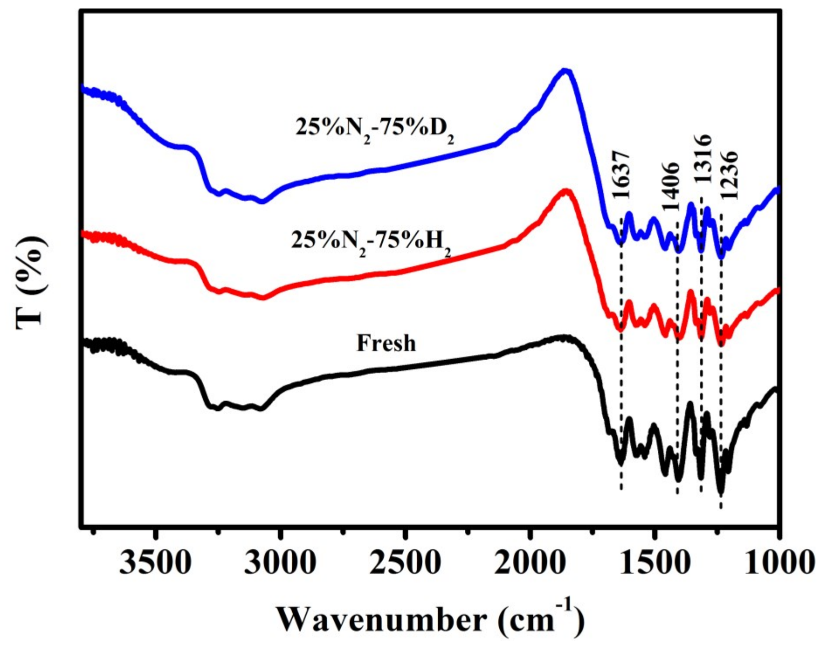

Fig. S32 C_3N_4 was firstly exposed to 25%N_2-75%H_2 or 25%N_2-75%D_2 atmospheres at 400 ^oC and 1 MPa, and then IR spectra of used C_3N_4 were collected.

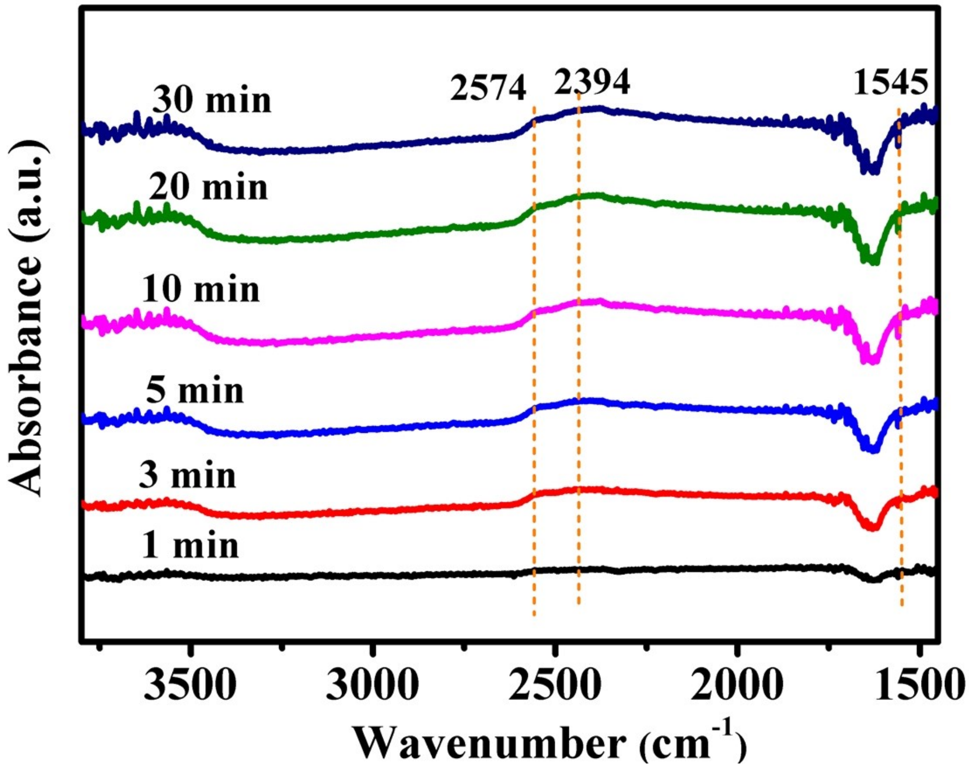

Fig. S33 Deuterium labeling: *in situ* DRIFTS experiments of RuCo DSAC after exposure to 25%N_2-75%D_2 mixture at 400 ^oC.

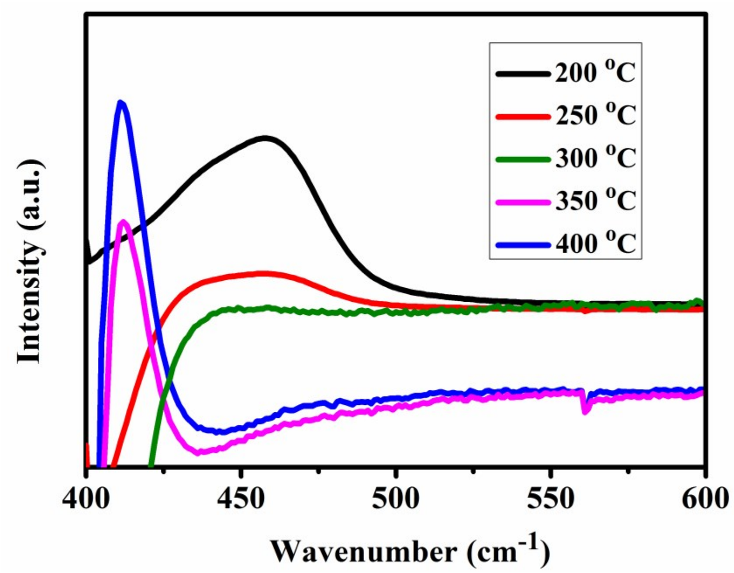

Fig. S34 UV-vis DRS spectra of RuCo DSAC catalyst upon the NH_3 synthesis reaction. We installed a flask trap including sulfur acid solution and para-(dimethylamino) benzaldehyde at the exit of our reactor during NH_3 synthesis reaction. The collected solution was then used for the UV-vis measurement.

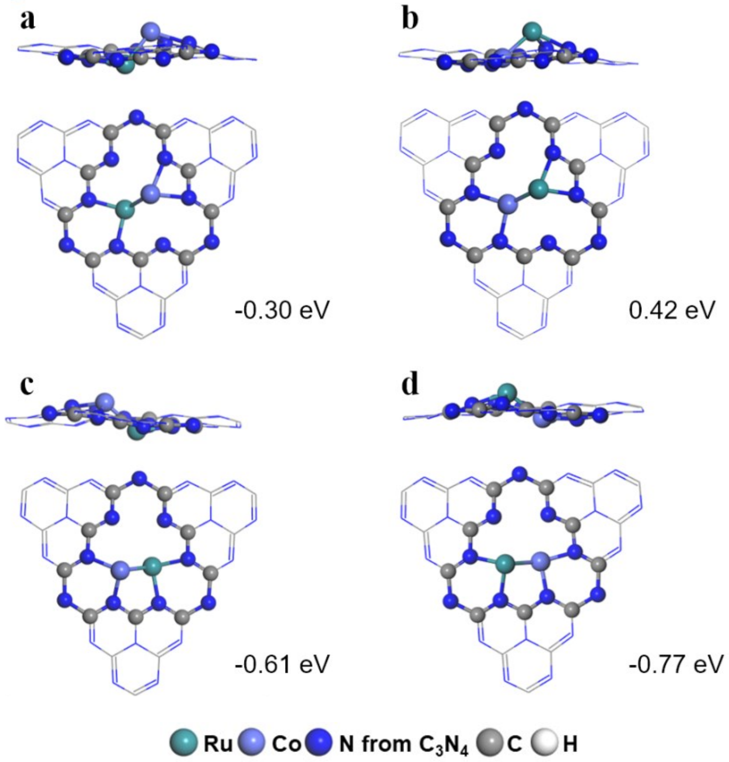

Fig. S35 Structures and energies of Ru-Co DSAC sites on g-C_3N_4 optimized by DFT calculation.

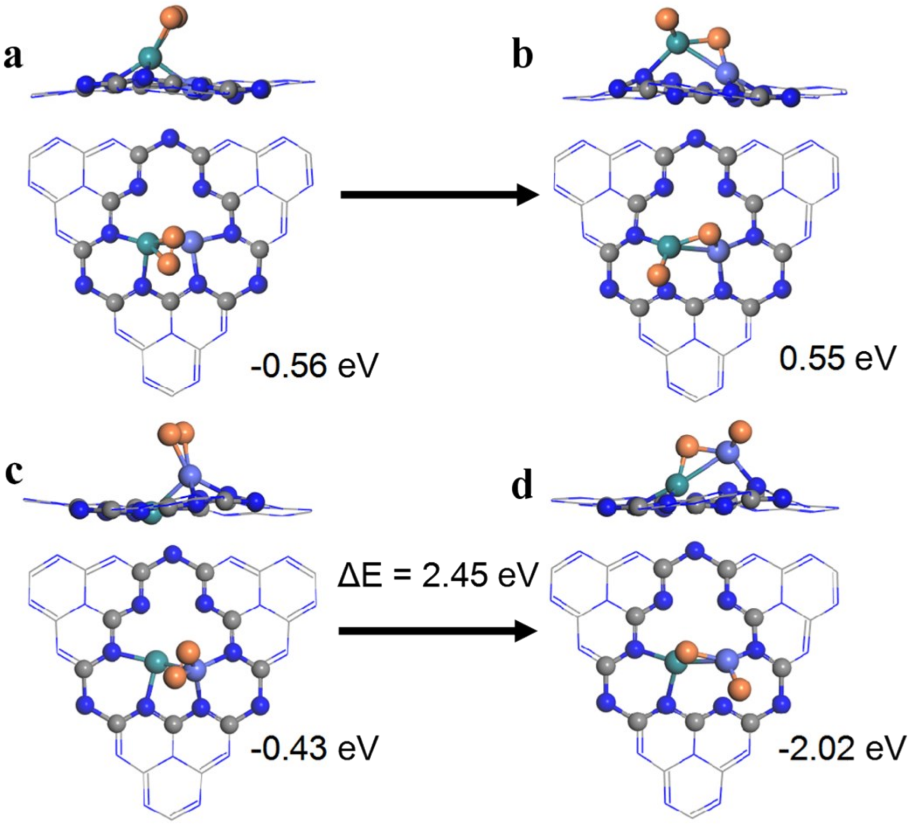

Fig. S36 a-d. Optimized structures and energies of adsorbed N_2 on **a)** Ru and **c)** Co. Dissociated of N_2 on **b)** Ru and **d)** Co on RuCo DSAC site.

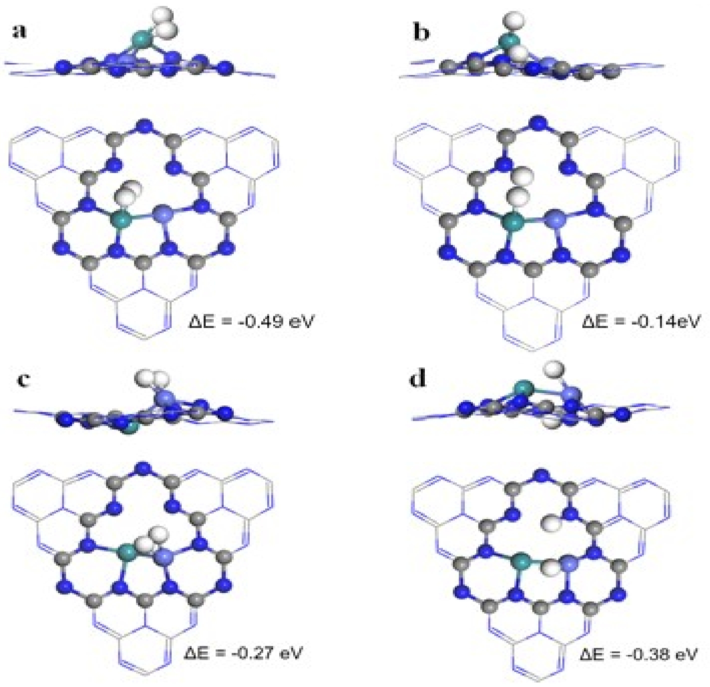

Fig. S37 Optimized structures and energies of adsorbed and dissociated H_2 on RuCo DSAC site.

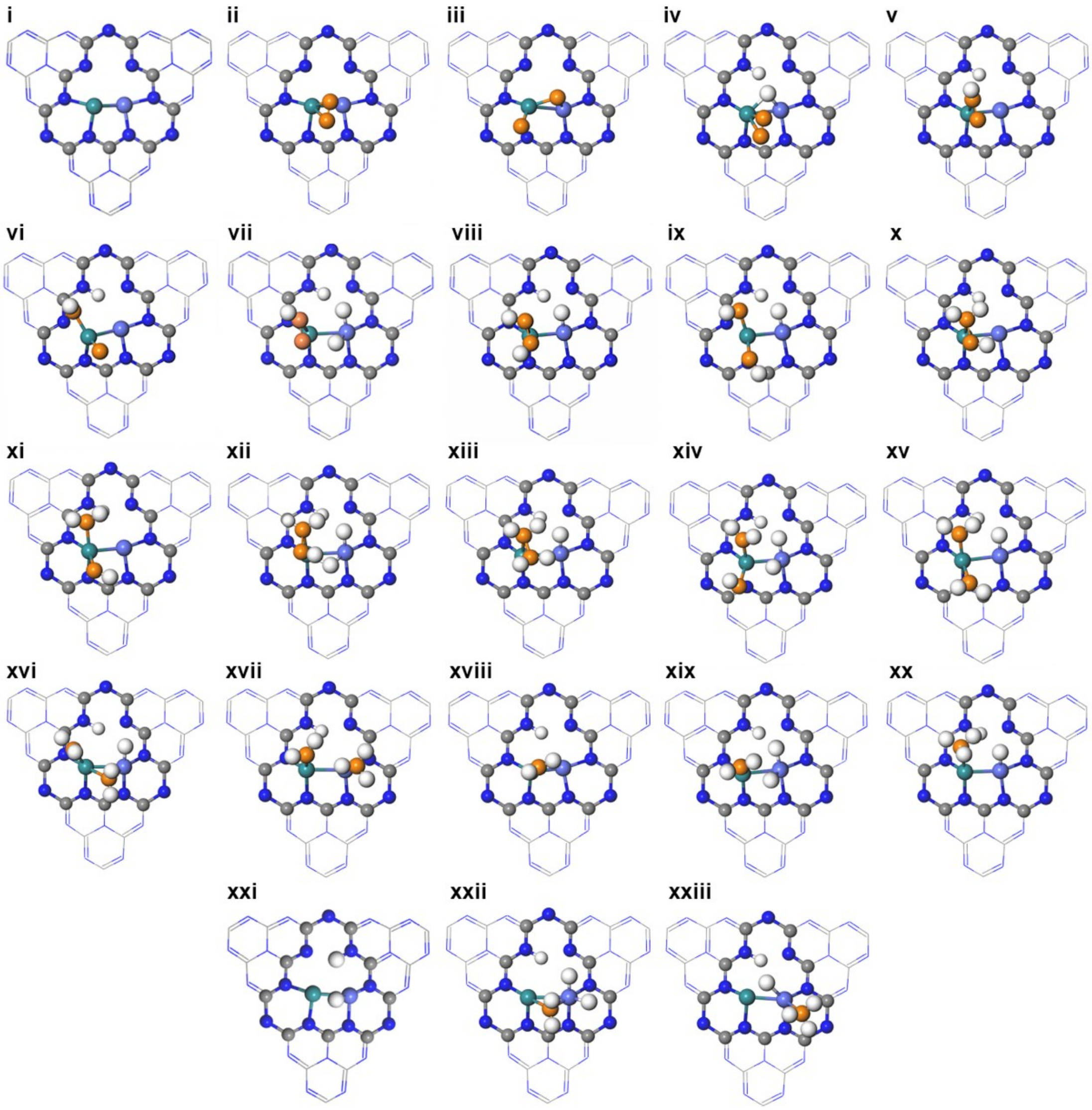

Fig. S38 Optimized structures in reaction pathway over RuCo DSAC site.

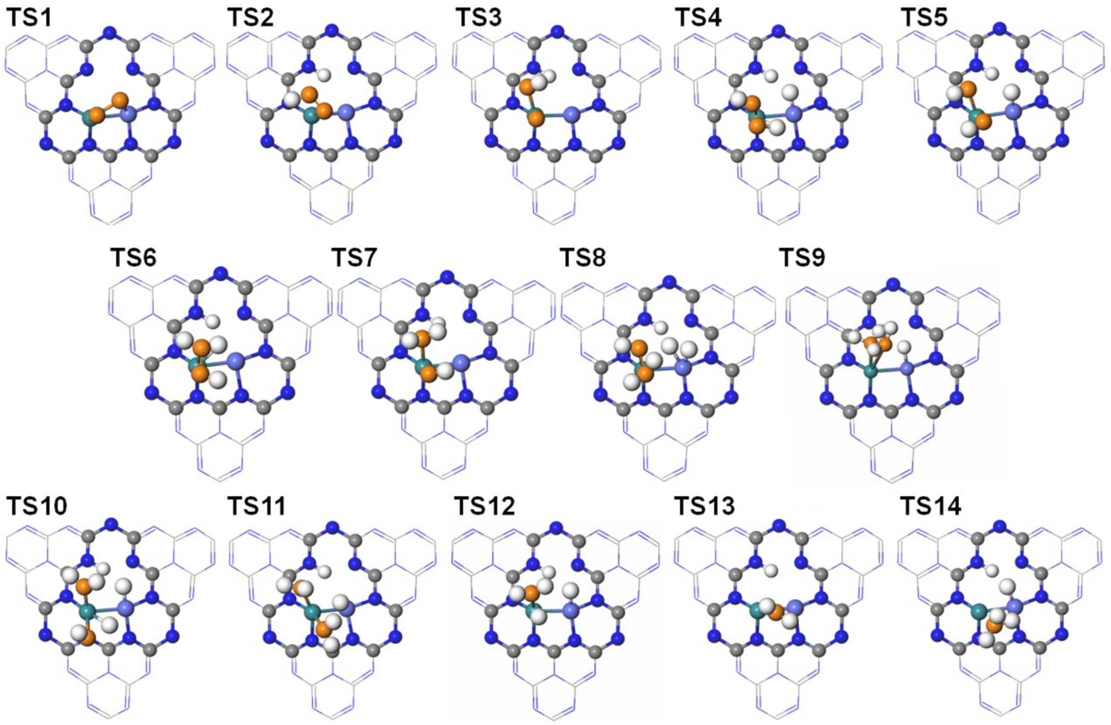

Fig. S39 Optimized structures of transition states for reaction pathway over RuCo DSAC site.

Table S1 ICP-AES data and textural properties of the as-prepared catalysts.

sample	Co content (wt.%)	Ru content (wt.%)	BET surface area (m^2/g)	Pore volume (cm^3/g)
Ru/N-C	-	1.34	88	0.33
Co SAC	2.05	-	180	0.55
RuCo DSAC	2.24	0.92	189	0.65

Table S2 EXAFS fitting parameters over as-prepared catalysts.

Sample	Path	C.N.	R (Å)	$\sigma^2 \times 10^3$ (Å^2)	ΔE (eV)	R factor
Co foil	Co-Co	12*	2.50±0.01	6.5±0.2	8.2±0.4	0.001
RuCo DSAC (Co K-edge)	Co-N	2.9±0.9	1.91±0.01	2.3±1.0	7.4±2.2	0.011
Co SAC	Co-N	2.7±0.6	1.89±0.01	4.0±1.5	3.2±2.6	0.014
Ru foil	Ru-Ru	12*	2.68±0.01	3.8±0.3	3.4±0.8	0.004
RuCo DSAC (Ru K-edge)	Ru-N	2.9±0.5	2.06±0.02	7.4±3.3	3.8±2.6	0.006

^{a}N: coordination numbers; ^{b}R: bond distance; $^{c}\sigma^2$: Debye-Waller factors; ^{d} ΔE_0: the inner potential correction. *R* factor: goodness of fit. * the experimental EXAFS fit of metal foil by fixing CN as the known crystallographic value.

Table S3 Surface Ru and Co composition over RuCo DSAC before and after Ar^{+} etching.

Sample	Surface Ru (%)		Surface Co (%)
	Before etching	After etching	
RuCo DSAC	0.90	No Ru signal detected	1.65

Table S4 d-band center of Co and Ru in bulk metal and RuCo DSAC.

N species	Co in Co SAC (e)	Co in RuCo DSAC (e)	Ru in RuCo DSAC (e)
Graphitic N	0.08	0.82	0.09
Pyridinic N	0.99	0.91	0.16
Pyrrolic N	1.08	0.97	0.45

Table S5 *d*-band center of Co and Ru in bulk metal and RuCo DSAC.

d-band	α-spin (eV)	β-spin (eV)	Average (eV)
Bulk-Co	-2.10	-0.49	-1.30
Co in RuCo DSAC	-2.18	-0.85	-1.65
Bulk Ru	-1.49	-1.49	-1.49
Ru in RuCo DSAC	-1.70	-1.73	-1.71

Table S6 NH_3 synthesis performance over Ru- and Co-based catalysts.

Sample	Ru loading (wt%)	Co loading (wt%)	Reaction Conditions T (°C)	P (MPa)	WHSV ($mL·g^{-1}·h^{-1}$)	NH_3 synthesis rate ($mmol_{NH3}\,g_{cat}^{-1}\,h^{-1}$)	TOF_{Ru} ($10^{-3}\,s^{-1}$)	TOF_{Co} ($10^{-3}\,s^{-1}$)	E_a ($kJ\,mol^{-1}$)	Ref.
RuCo DSAC	0.92	2.24	200	1.0	60 000	1.24	1.80	0.30	58	This work
			350	1.0	60 000	3.8	17.0	2.80		
Ba/RuCo DSAC	0.92	2.24	300	1.0	60 000	4.10			-	This work
			350	1.0	60 000	6.82			-	
5wt%Cs-Ru/C	1.34	-	200	1.0	60 000	0.09	-	-	-	This work
Co-N-C	-	3.73	350	1.0	60 000	4.34	4.53	-	-	4
Ru/Y_5Si_3	7.80	-	400	0.1	18 000	1.90	0.07	-	52	5
$Ru/C12A7:e^-$	4	-	400	1.0	18 000	6.09	4.30	-	-	6
Ru-Ba/AC	9.10	-	400	1.0	18 000	8.29	2.60	-	-	6
$Ru–Ba/Al_2O_3$ -980	5	-	400	1.0	60 000	7.22	4.50	-	103	7
Ba-Ru SAs/S-1	0.23	-	400	0.1	18 000	1.39	16.96	-	90.5	8
Cs-Ru/MgO	0.27	-	375	0.1	18 000	$4.6*10^{-3}$	0.05	-	100.9	8
$Ru/BaO-CaH_2$	10	-	340	0.1	18 000	10.50	0.009	-	41	9
LaCoSi	-	26.10	400	0.1	36 000	1.25		0.08	42	10
$Co/C12A7:e^-$	-	2.60	400	0.1	18 000	1.76		$4.1*10^{-3}$	49.5	11
Ba–Co/AC	-	10	400	0.1	18 000	0.10		$0.13*10^{-3}$	98.3	11
Co_3Mo_3N	-	35.20	500	0.1	12 000	0.49		-	-	12
Co/C	-	-	400	1.0	96 000	0.72		-	149	13
BaH_2-Co/CNTs	-	5.20	300	1.0	60 000	4.80		1.51	58	14
BaO-Co/CNTs	-	3.25	300	1.0	60 000	0.03		0.02	136	14
$Cs–Co_3Mo_3N$	-	36.90	400	0.1	9000	0.97		-	57	15

References

1. L. F. Mattheiss and R. E. Dietz, *Phys. Rev. B.*, 1980, **22**, 1663–1676.

2. D. H. Pearson, C. C. Ahn and B. Fultz, *Phys. Rev. B.*, 1993, **47**, 8471–8478.

3. H. Ouyang and J.-T. Kwan, *J. Appl. Phys.*, 2002, **92**, 7510–7513.

4. X. Wang, X. Peng, W. Chen, G. Liu, A. Zheng, L. Zheng, J. Ni, C. Au and L. Jiang, *Nat. Commun.*, 2020, 11, 653.

5. Y. Lu, J. Li, T. Tada, Y. Toda, S. Ueda, T. Yokoyama, M. Kitano and H. Hosono, *J. Am. Chem. Soc.*, 2016, **138**, 3970–3973.

6. M. Kitano, Y. Inoue, Y. Yamazaki, F. Hayashi, S. Kanbara, S. Matsuishi, T. Yokoyama, S. W. Kim, M. Hara and H. Hosono, *Nat. Chem.*, 2012, **4**, 934–940 .

7. B. Lin, L. Heng, B. Fang, H. Yin, J. Ni, X. Wang, J. Lin and L. Jiang, *ACS Catal.*, 2019, **9**, 1635−1644 .

8. J-Z. Qiu, J. Hu, J. Lan, L-F. Wang and J. Jiang, *Chem. Mater.*, 2019, **31**, 9413−9421.

9. M. Hattori, T. Mori, T. Arai, Y. Inoue, M. Sasase, T. Tada, M. Kitano, T. Yokoyama, M. Hara, and H. Hosono, *ACS Catal.*, 2018, **8**, 10977-10984.

10. Y. Gong , J. Wu, M. Kitano, J. Wang, T-N. Ye, J. Li, Y. Kobayashi, K. Kishida, H. Abe, Y. Niwa, H. Yang, T. Tada and H. Hosono, *Nat. Catal.*, 2018, **1**, 178–185.

11. Y. Inoue, M. Kitano, M. Tokunari, T. Taniguchi and H. Hosono, *ACS Catal.*, 2019, **9**, 1670–1679.

12. I. Alshibane, A. Daisley, J. S. J. Hargreaves, A. L. Hector, S. Laassiri, J. Rico and R. I. Smith, *ACS Sustain. Chem. Eng.*, 2019, **5**, 9214–9222.

13. S. Hagen, R. Barfod, R. Fehrmann, C. J. H. Jacobsen, H. T. Teunissen, K. Stahl and I. Chorkendorff, *Chem. Commun.*, 2002, **11**, 1206–1207.

14. W. Gao, P. Wang, J. Guo, F. Chang, T. He, Q. Wang, G. Wu and P. Chen, *ACS Catal.*, 2017, **7**, 3654–3661.

15. R. Kojima and K. Aika, *Appl. Catal. A.*, 2001, **218**, 121–128.
